# *Staphylococcus aureus* cell wall structure and dynamics during host-pathogen interaction

**DOI:** 10.1371/journal.ppat.1009468

**Published:** 2021-03-31

**Authors:** Joshua A. F. Sutton, Oliver T. Carnell, Lucia Lafage, Joe Gray, Jacob Biboy, Josie F. Gibson, Eric J. G. Pollitt, Simone C. Tazoll, William Turnbull, Natalia H. Hajdamowicz, Bartłomiej Salamaga, Grace R. Pidwill, Alison M. Condliffe, Stephen A. Renshaw, Waldemar Vollmer, Simon J. Foster

**Affiliations:** 1 Department of Molecular Biology and Biotechnology, University of Sheffield, Western Bank, Sheffield, United Kingdom; 2 The Florey Institute for Host-Pathogen Interactions, University of Sheffield, Sheffield, United Kingdom; 3 Biosciences Institute, Newcastle University, Newcastle upon Tyne, United Kingdom; 4 Centre for Bacterial Cell Biology, Biosciences Institute, Newcastle University, Newcastle upon Tyne, United Kingdom; 5 The Bateson Centre, University of Sheffield, Firth Court, Western Bank, Sheffield, United Kingdom; 6 Department of Biomedical Science, University of Sheffield, Firth Court, Western Bank, Sheffield, United Kingdom; 7 Department of Infection, Immunity and Cardiovascular Diseases, University of Sheffield, Beech Hill Road, Sheffield United Kingdom; University of Tubingen, GERMANY

## Abstract

Peptidoglycan is the major structural component of the *Staphylococcus aureus* cell wall, in which it maintains cellular integrity, is the interface with the host, and its synthesis is targeted by some of the most crucial antibiotics developed. Despite this importance, and the wealth of data from *in vitro* studies, we do not understand the structure and dynamics of peptidoglycan during infection. In this study we have developed methods to harvest bacteria from an active infection in order to purify cell walls for biochemical analysis *ex vivo*. Isolated *ex vivo* bacterial cells are smaller than those actively growing *in vitro*, with thickened cell walls and reduced peptidoglycan crosslinking, similar to that of stationary phase cells. These features suggested a role for specific peptidoglycan homeostatic mechanisms in disease. As *S*. *aureus* missing penicillin binding protein 4 (PBP4) has reduced peptidoglycan crosslinking *in vitro* its role during infection was established. Loss of PBP4 resulted in an increased recovery of *S*. *aureus* from the livers of infected mice, which coincided with enhanced fitness within murine and human macrophages. Thicker cell walls correlate with reduced activity of peptidoglycan hydrolases. *S*. *aureus* has a family of 4 putative glucosaminidases, that are collectively crucial for growth. Loss of the major enzyme SagB, led to attenuation during murine infection and reduced survival in human macrophages. However, loss of the other three enzymes Atl, SagA and ScaH resulted in clustering dependent attenuation, in a zebrafish embryo, but not a murine, model of infection. A combination of *pbp4* and *sagB* deficiencies resulted in a restoration of parental virulence. Our results, demonstrate the importance of appropriate cell wall structure and dynamics during pathogenesis, providing new insight to the mechanisms of disease.

## Author summary

The prevalence of methicillin resistant *Staphylococcus aureus* (MRSA) in both hospitals and the wider community places a huge weight on healthcare providers. To discover new control regimes, it is therefore important to understand how the pathogen behaves within the relevant environment of the host. This is often hampered by the ability to obtain sufficient *ex vivo* pathogen samples for study. We have developed a method to isolate *S*. *aureus* from the infected host to be able to analyse cellular morphology and structure. *S*. *aureus*, isolated from an infected kidney abscess are smaller in size, with thicker cell walls than exponentially growing cells *in vitro*. Their cell wall peptidoglycan also is less crosslinked. These features suggested the role of components controlling cell wall homeostasis as being important for infections. We tested the role of PBP4, known to increase cell wall crosslinking and found a *pbp4* mutant to have increased survival in macrophages and fitness within the murine host. Conversely the peptidoglycan hydrolase SagB, whose loss results in thinner cell walls was attenuated in the murine systemic model of infection, with concomitant loss of fitness within macrophages. Our study reveals an important adaptation to the host environment and the role of those components involved in cell wall homeostasis *in vivo*.

## Introduction

*Staphylococcus aureus* is adaptable, it exists as a commensal, colonising the nares and skin of around 30% humans [1], but also has the capacity to be a serious opportunist pathogen, often through injury or medical intervention [2]. Upon entering a host, *S*. *aureus* can infect a wide repertoire of sites around the body, causing skin and soft tissue infection (SSTIs), sepsis, osteomyelitis, peritonitis, pneumonia and endocarditis [3]. Also, infections are inherently dynamic as they progress from establishment, to resolution or potentially host mortality [4]. Such versatility is manifested by physiological adaptation to a changing environment. The cell wall forms the interface between *S*. *aureus* and the human host, where it not only is essential for viability of the bacterium but remains dynamic during growth and interacts with the host immune system. Despite this importance, the structure and dynamics of the cell wall during infection are poorly understood.

Peptidoglycan (PG) is the major component of the cell wall and surrounds the cytoplasmic membrane as a single macromolecule, forming the sacculus [5]. PG consists of glycan chains made of alternating *N*-Acetylmuramic acid (Mur*N*Ac) and *N-*Acetylglucosamine (Glc*N*Ac) residues which are connected by short peptides that are peptides bound to the Mur*N*Ac residues [6]. The nascent stem peptides in *S*. *aureus* PG consists of l-alanine, d-*iso*glutamine, l-lysine with a penta-glycine attached to the epsilon amino group, and a terminal d-alanine-d-alanine [7,8].

Crosslinks in PG produce a three-dimensional mesh with strength and rigidity [9]. In *S*. *aureus*, up to 80–90% of the stem peptides are linked by a 3–4 crosslink via the pentaglycine bridge [10]. *S*. *aureus* PG has a reduced crosslinking in stationary phase compared to exponential phase when grown *in vitro* in synthetic medium, which could be due to glycine depletion [11]. PG can also be modified after its synthesis by O-acetylation of Mur*N*Ac residues [12], which in *S*. *aureus* contributes to lysozyme resistance and pathogenicity [13,14].

PG plays an important role in host-pathogen interactions, with host receptors including nucleotide-binding oligomerisation domain protein (NOD) 1 and NOD2 recognising specific PG motifs. Detection of muropeptides by NOD1 or 2 results in the secretion of cytokines such as interleukin (IL) 6 and tumour necrosis factor alpha (TNF-α) [15], and the activation of the NF-κB pro-inflammatory cascade [16] causing the transcription of pro-inflammatory cytokines and leukocyte recruitment to help to clear bacterial infection [17]. PG can also augment *S*. *aureus* infection when co-administered in a murine sepsis model of infection [18].

Growth and cell division require both PG synthases and hydrolases [5]. Penicillin binding proteins (PBPs) complete the final stages of PG synthesis, performing the glycosyltransferase and/or the transpeptidase reactions required to synthesize nascent PG and incorporate it into the sacculus [19]. *S*. *aureus* possesses 4 native PBPs. The transpeptidase PBP1 is essential for growth [20] and division septa formation [21,22]. PBP2 is essential and has glycosyltransferase and transpeptidation activities [23]. The non-essential PBP3 is a monofunctional transpeptidase [24] interacting with RodA for the correct localisation at mid-cell and the insertion of PG at sites other than the septum [25]. PBP4 is responsible for the high cross-linkage in the PG of *S*. *aureus* [26] consistent with its high activity *in vitro* [27]. PBP4 is required for β-lactam resistance in community-acquired MRSA strains [28]. A *pbp4* mutant caused larger skin lesions, without increased bacterial load, compared to a wildtype *S*. *aureus* in a murine skin abscess model of infection [29], suggesting an increased virulence from exacerbated disease presentation. This was hypothesised to be caused by an increased production of host IL-1β [29]. MRSA strains also encode the non-native *mecA* gene, encoding PBP2A, responsible for low level β-lactam antibiotic resistance, or high level when present with specific *rpoB* and *rpoC* mutations [30]. *S*. *aureus* making PBP2A produces PG with a low level of crosslinking, similar to that of *pbp4* mutants, when all native PBPs are inactivated by the presence of β-lactam antibiotic. This indicates that PBP2A can produce a basal level of PG cross-links to facilitate cell survival in the presence of β-lactams, but it cannot complement for the loss of PBP4 function, resulting in low PG cross-linkage [29]. Mice infected with MRSA and then treated with β-lactam antibiotics (therefore producing bacteria with poorly crosslinked PG) developed significantly larger lesions in comparison to mice treated with PBS [29]. The MRSA strain *S*. *aureus* COL is viable with only PBP1 and PBP2 with no impact on cell growth or morphology, but is attenuated in a *Drosophila* model of infection compared to a wildtype COL strain [31].

*S*. *aureus* has multiple PG hydrolases allowing the cleavage of the glycan backbone and sidechains. *N*-acetyl-β-d-muramidases (muramidases, including the subset of lytic transglycosylases) cleave after the *N*-acetylmuramic acid residue in the glycan backbone, whereas *N*-acetyl-β-d-acetylglucosaminidases (glucosaminidases) cleave after the N-acetylglucosamine moiety [32]. The genome of *S*. *aureus* encodes for two putative lytic transglycosylases (IsaA and SceD) [33], as well as four putative glucosaminidases (Atl, SagB, SagA and ScaH) [34,35]. *S*. *aureus* Atl is bifunctional, containing both a cell wall amidase and a glucosaminidase domain [36,37]. Atl is the major autolysin of *S*. *aureus* and is involved in the separation of daughter cells, demonstrated by the formation of clusters in *atl* deletion mutants [37], but this does not impact upon virulence [38]. A more recent study has shown that the loss of glucosaminidase results in clustering and attenuation in a murine model of osteomyelitis [39]. The glucosaminidase SagB is responsible for cleaving glycans to their mature length [34,35], without which cells show morphological and growth defects, as well as aberrant protein secretion [34,35]. Mutants with inactive *sagA* or *scaH* genes show similar cell architecture, growth and cell division to wildtype *S*. *aureus* [34,35]. Mutants lacking up to three of the four glucosaminidases are viable [35].

While much is known about the structure of *S*. *aureus* PG grown *in vitro* [40], there is, as of yet, no data on the structure during an infection in any organism. Several studies have utilised “pseudo *in vivo*” conditions to mimic the complex environment of an infection. *Staphylococcus epidermidis* PG isolated from an *in vitro* biofilm in platelet concentrates show substitutions in the pentaglycine bridge, with glycine being substituted for serine or alanine residues, as well as increased O-acetylation, providing resistance to lysostaphin and lysozyme [14,41].

Here we further developed reverse phase high performance liquid chromatography (RP-HPLC) coupled to mass spectrometry (MS) methodologies, with MS/MS allowing the identification of a muropeptide based on its mass and fragmentation pattern [42], to determine the PG composition during a murine sepsis model of infection [4,18]. This provides the first data of PG structure during an active infection revealing the impact of cell wall homeostatic mechanisms on *S*. *aureus* virulence.

## Results

### Morphological analysis of *S*. *aureus* during infection

**Fig 1:**
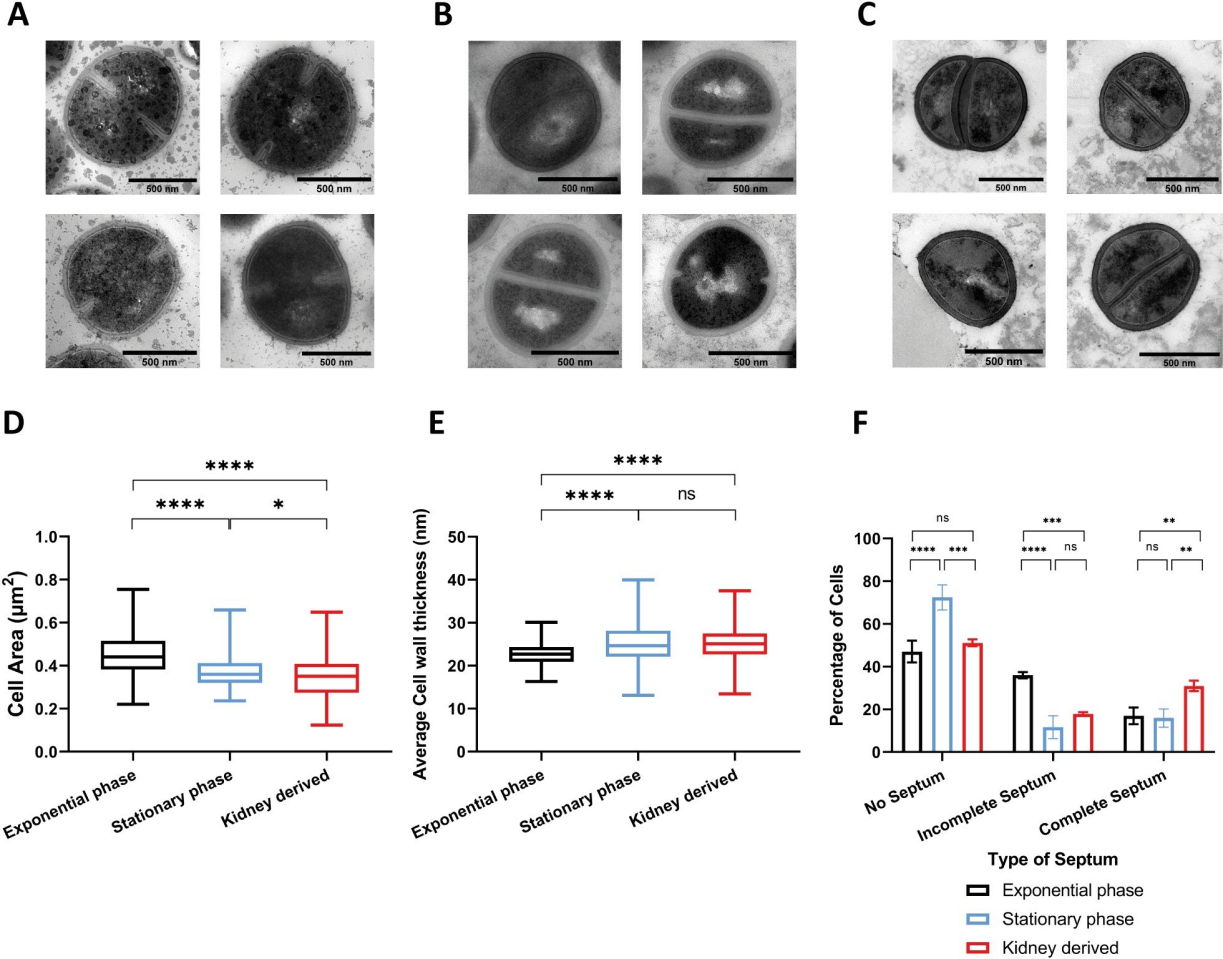
TEM analysis of *S*. *aureus* NewHG grown *in vitro* and *in vivo*. Thin sections of chemically fixed *S*. *aureus* NewHG *kan*^*R*^ (SJF 3680) cultured in TSB to **(A)** exponential or **(B)** stationary phase or **(C)** recovered from murine kidneys (72 hpi). Scale bars (black line) represent500 nm. **(D)** Cell area of NewHG *kan*^*R*^ cultured in TSB to exponential phase (3 independent repeats totalling 411 cells), in TSB to stationary phase (3 independent repeats totalling 320 cells) or recovered from murine kidneys (72 hpi) (2 independent repeats totalling 180 cells). Results analysed using a one-way ANOVA with multiple comparisons (* p = 0.0364, **** p < 0.0001). **(E)** Average cell wall thickness of NewHG *kan*^*R*^ cultured in TSB to exponential phase (black lines, 3 independent repeats totalling 367 cells), in TSB to stationary phase (blue lines, 3 independent repeats totalling 256 cells) or recovered from murine kidneys (72 hpi) (red lines, 2 independent repeats totalling 104 cells). Results analysed using a one-way ANOVA with multiple comparisons (**** p < 0.0001). Box and whiskers for **(D)** and **(E)** represent mean, lower and upper quartiles, and range respectively. **(F)** Normalised percentage of NewHG *kan*^*R*^ that show no, incomplete or a completed septum. Normalised proportions were compared using a two-way ANOVA with multiple comparisons with Tukey’s correction (no septa *** p = 0.0001, **** p < 0.0001; incomplete septa *** p = 0.0007, **** p < 0.0001; complete septa ** p = 0.0034 and 0.0057). Error bars represent the standard deviation of the mean. (Exponential phase cultured in TSB–black bars, stationary phase cultured in TSB–blue bars, cells recovered from murine kidney homogenate–red bars).

The murine sepsis model of infection [4,18] was used to derive *in vivo* grown *S*. *aureus* cells. This model has an organ tropism that leads to kidney abscesses, providing a concentration of bacterial cells for analysis [4]. 72 hours post infection (hpi) kidneys were harvested and homogenised. For transmission electron microscopy (TEM), bacteria were recovered from the homogenate by centrifugation, processed for analysis and compared to *in vitro* derived cells. TEM was performed on three independent *S*. *aureus* NewHG *kan*^*R*^ exponential phase (OD_600_ of 0.6) or stationary phase (8 hours of growth, OD_600_ of around 9–10) cultures (Figs 1A, 1B, S1A and S1B) and two independent *S*. *aureus* NewHG *kan*^*R*^ samples recovered from murine kidneys (Figs 1C and S1C). Uninfected kidneys were also processed as a control (S1D Fig).

Initially, cell area was compared (Fig 1D). *S*. *aureus* cells grown to exponential phase have a significantly greater cell area (0.45 μm^2^ ± 0.09 μm^2^) than those cultured to stationary phase (0.37 μm^2^ ± 0.06 μm^2^) in TSB (p < 0.0001), as has been previously shown [43]. *S*. *aureus* cells recovered from murine kidneys also show a significant reduction in area (0.35 μm^2^ ± 0.1 μm^2^) compared to both exponential and stationary phase cells (p < 0.0001 and p = 0.0364, respectively).

The *S*. *aureus* cell wall forms a distinct layer around the periphery of the cell (Fig 1E). Exponential phase cells have a significantly thinner cell wall (22.7 nm ± 2.4 nm) than both stationary phase cells (25.1 nm ± 4.5 nm, p < 0.0001) and those from kidneys (25.3 nm ± 4.3 nm, p < 0.0001). No significant difference between cell wall thickness of stationary phase cells or cells recovered from kidneys could be detected (p = 0.9611).

The above analysis reveals that *in vivo*, within 3-day infected kidneys, the bacteria have a morphology reminiscent of quiescent cells [43]. To determine if active cell division is taking place the percentage of *S*. *aureus* cells possessing, or lacking, incomplete or complete septa was determined for each growth condition (Fig 1F). A significantly greater proportion of stationary phase cells had no septa compared to both exponential phase and *in vivo* derived cells (p < 0.0001), the latter 2 groups not showing a significant difference (p = 0.5429), suggesting that *in vivo* cells divide more often or slower. However, exponential phase cells have a significantly greater proportion with incomplete septa than both stationary phase and *in vivo* cells (p < 0.0001 and p = 0.0007) and kidney derived cells (p = 0.0007), the latter 2 groups not showing a significant difference (p = 0.2617). *In vivo* there was a significantly greater proportion of undivided cells with a complete septum than exponential phase (p = 0.0057) and stationary phase cells (p = 0.0034), the latter 2 groups did not show a significant difference (p = 0.9548). All together these data point to possible alterations in cell wall structure and dynamics *in vivo*, with thicker cell walls and reduced cell separation after septation.

### Infection-associated *S*. *aureus* PG structural analysis

**Fig 2:**
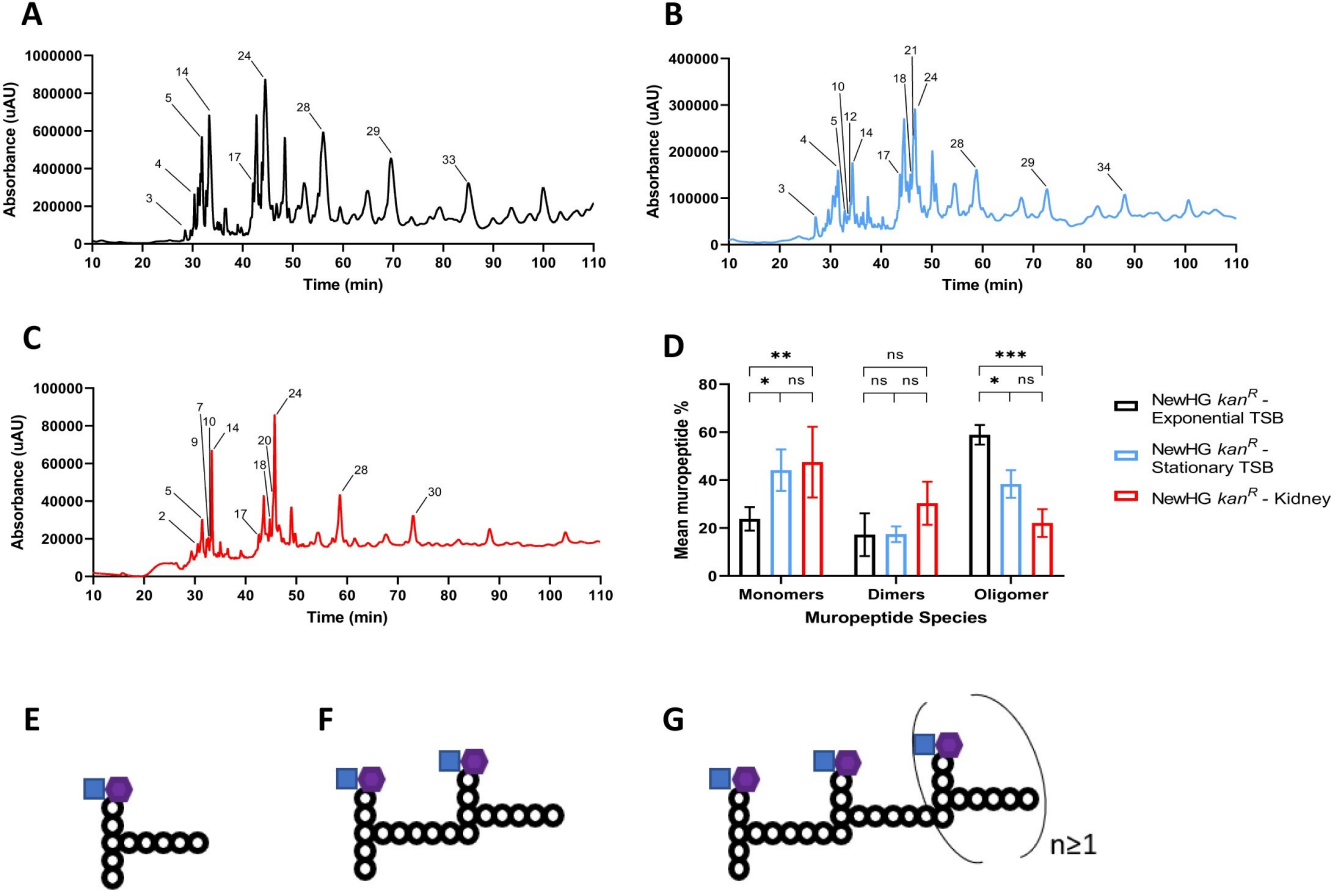
Analysis of *S*. *aureus* muropeptide profiles in different growth conditions. Representative muropeptide profiles of NewHG *kan*^*R*^ (SJF 3680) cultured in TSB in **(A)** exponential phase (OD_600_ ~0.6) or **(B)** stationary phase (OD_600_ ~9). Muropeptides have been labelled after being identified by MS (S1 Table) **(C)** Representative muropeptide profile of NewHG *kan*^*R*^ (SJF 3680) recovered from murine kidneys 72 hpi. Identified muropeptides are labelled using muropeptide numbers from S3 Table. **(D)** Area of eluted identified UV-absorbing peaks shown as a percentage of the total of all identified peaks grouped based on cross-linking (exponential phase TSB—black bars n = 3, stationary phase TSB–blue bars n = 3, NewHG *kan*^*R*^ recovered from murine kidney–red bars, n = 2 independent experiments, each consisting of 10 mice, error bars represent the standard deviation of the mean). A two-way ANOVA with Sidak’s multiple comparison post-test was used to compare abundance of each muropeptide species (monomers * p = 0.0108, ** p = 0.0081; oligomers * p = 0.0101, *** p = 0.0002). Representative generic muropeptide structures, **(E)** monomeric, **(F)** dimeric and **(G)** oligomeric.

Morphological analysis revealed that *S*. *aureus* recovered from murine kidneys resemble quiescent cells, being smaller with thickened cell walls (Fig 1). The composition of the PG was determined by muropeptide analysis [42,44,45], comparing *in vivo* derived PG to that from *in vitro* grown exponential and stationary phase cells (Fig 2A and 2B). The traces show the identification of monomeric muropeptide species (muropeptides 3, 4, 5, 10, 12 and 14), dimeric species (muropeptides 17, 18, 21 and 24) and higher crosslinked trimeric (muropeptide 28), tetrameric (muropeptide 29) and pentameric (muropeptides 33 and 34) species as described in S3 Table.

Kidney homogenates from 2 independent experiments (10 mice in each experiment, totalling 20 kidneys) were combined and PG was collected and purified, which equated to the material from 1.03 x 10^8^ and 9.3 x 10^7^ CFU, respectively. After purification, the remaining pellet appeared grey rather than the white colour observed from *in vitro* samples. Further SDS washes or pronase treatments did not alter the pellet. However, the presence of the host kidney derived material did not prevent mutanolysin digestion of the PG. A representative muropeptide profile of PG from *S*. *aureus* NewHG purified from murine kidneys is shown in Fig 2C. Monomeric, dimeric, trimeric, and tetrameric muropeptide species were identified, which all corresponded to those found from *in vitro* growth (S4A–S4H Table).

The area of each identified UV-absorbance peak was calculated, expressed as a percentage of the total area of all identified peaks and grouped based on the level of crosslinking (Fig 2D), as derived from monomeric (Fig 2E), dimeric (Fig 2F) and oligomeric (Fig 2G) material. Significantly more monomeric muropeptides are found in stationary phase and *in vivo* samples (p = 0.0180 and p = 0.0081, respectively) than exponential phase cells, the former 2 groups not showing a significant difference (p = 0.8734). No differences in dimer levels were found. Significantly fewer oligomeric species were present in stationary phase and *in vivo* samples compared to exponential phase cells (p = 0.0101 and p = 0.0002, respectively). The former 2 groups did not show a significant difference in oligomer levels (p = 0.0691). An increase in monomers and a concomitant decrease in oligomers evidences a decrease in PG cross-linking as cells enter stationary phase or are *in vivo* derived.

### The role of the PG synthase PBP4 in infection

**Fig 3:**
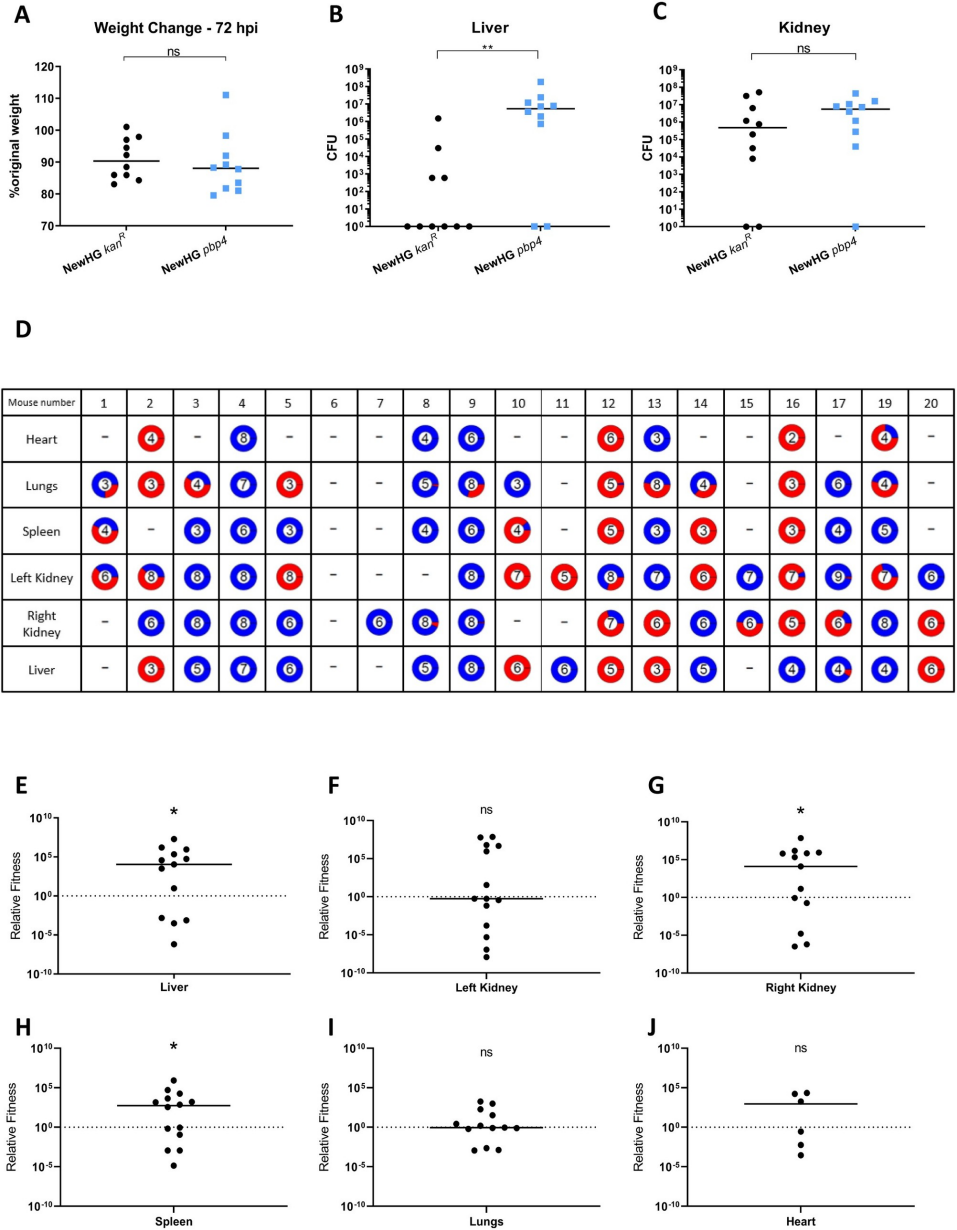
The role of PBP4 in *S*. *aureus* virulence. Mice (n = 10) were injected with approximately 5x10^6^ CFU *S*. *aureus* NewHG *kan*^*R*^ (WT, SJF 3680) or NewHG *pbp4*::*ery* (SJF 5103). **(A)** Weight loss 72 hpi and CFUs recovered from **(B)** livers, **(C)** kidneys were determined. Groups were compared using a Mann-Whitney U test (NewHG *kan*^*R*^–black circles, NewHG *pbp4*::*ery* blue squares) (** p = 0.0051, * p = 0.0410). **(D)** Mice (n = 20) were injected with a 1:1 ratio (totalling 7 x 10^6^ CFU) of NewHG *kan*^*R*^ (SJF 3680, red) and NewHG *pbp4*::*ery* (SJF 5103, blue). 72 hpi mice were culled and CFU ratios within organs were determined. The number in each pie chart represents the log total number of bacteria recovered (i.e. 10^6^ CFU = 6). Mice 9 and 12 were culled at 54 hpi due to severity limits but are included in the analysis. Mouse 18 was found dead at 72 hpi and is excluded from analysis. The relative fitness of NewHG *pbp4*::*ery* (SJF 5103) against NewHG *kan*^*R*^ (SJF 3680) was calculated using the formula $w=\frac{x_{2(1-x_{1})}}{x_{1(1-x_{2})}}$ (where *w =* relative fitness, *X*_*1*_ *=* starting mutant proportion and *X*_*2*_ *=* ending mutant proportion). This was calculated for the **(E)** liver (* p = 0.0105), **(F)** left kidney (p = 0.3258), **(G)** right kidney (* p = 0.0327), **(H)** spleen (* p = 0.0476), **(I)** lungs (p = 0.3396) and **(J)** heart (p = 0.43275). Line on graph depicts the median. Statistical significance was determined using a one sample Wilcoxon signed rank test, comparing the results to a theoretical median of 1, which would indicate an equal fitness between the strains.

Reduced *in vivo* oligomeric PG suggests a downregulation of crosslinking activity, which PBP4 is known to be responsible for *in vitro* [26], thus the role of PBP4 in infection was investigated. To determine the role of PG dynamics in infection, both the zebrafish and murine sepsis models were used. Comparing NewHG *pbp4*::*ery* (SJF 5103)to its parent NewHG in the zebrafish model of infection revealed no significant difference in larval mortality rate (S2A Fig). In the murine sepsis model, using an inoculum of 5 x 10^6^ or 1 x 10^7^ CFU/mouse no significant differences were observed for weight loss or kidney CFU when comparing both strains at the different inocula (Figs 3A and S2B). The *pbp4* strain gave a significantly greater recovery of bacteria from the liver, 3 days post infection (Figs 3B and S2C: p = 0.0051 and p = 0.0294 respectively), suggesting that *S*. *aureus pbp4* mutant is fitter in the murine liver than the parental strain. In the murine sepsis model bacteria are initially captured by Kupffer cells in the liver, from whence kidney abscesses are seeded [4]. During this process, an immunological bottleneck occurs in the phagocytes that results in clonal expansion. To determine if the increased liver CFU associated with the *pbp4* mutation is due to a bypass of the immune bottleneck, matched *pbp4* strains, isogenic apart for antibiotic resistance markers (to allow selection), were created. Mice were injected with a 1:1 ratio of marked strains (total 7 x 10^6^ CFU) and infection dynamics monitored over time (S2 Fig). As expected, after infection the bacteria are found primarily in the liver and spleen (S2G and S2J Fig). Within 24 hrs CFU begin to increase in the kidneys, associated with the formation of characteristic abscesses (S2H and S2I Fig), whilst numbers remain low in the lungs and heart throughout (S2K and S2L Fig). Clonal expansion is observed by an alteration in the ratio of the matched strains over time (S2M–S2R Fig), as measured by population evenness, from evenly mixed populations to those dominated by a single clone [4]. By 72 hpi predominantly clonal organs are observed (single colour dominating; S2E and S2F Fig). Linear regression revealed a statistically significant decrease in population evenness in the liver for both *pbp4* (p = 0.0036, F = 11.17, R^2^ = 0.3829) and its parent (p = 0.0448, F = 4.795, R^2^ = 0.2422) and no difference between the 2 strains (p = 0.6077, F = 0.2686) demonstrating both strains pass through the immune bottleneck similarly. Overall, this suggests that *S*. *aureus pbp4* mutant is fitter in the murine liver than the parental strain.

To confirm the proposed fitness phenomenon, 20 mice were injected with a 1:1 ratio (totalling 7 x 10^6^ CFU) of NewHG and NewHG *pbp4*. Mice were infected for a period of 72 hours before being culled and organs harvested. The total number, and ratio of each strain, recovered from each organ was calculated (Fig 3D). The pie charts show the ratio of NewHG (red) to NewHG *pbp4* (blue), with the central number indicating the log total number of bacteria recovered, with most organs dominated by a single strain at 72 hpi.

To determine if the ratios of the recovered strains are significantly different from the infectious dose (1:1), the relative fitness of NewHG *pbp4* was calculated using the formula *w* = x_2_(1-x_1_) /x_1_(1-*x*_2_) (where w = relative fitness, x_1_ = starting mutant proportion and x_2_ = ending mutant proportion) [46]. This gives a numerical value for the relative fitness of NewHG *pbp4*, where a value of 1 indicates an equal fitness between strains, a value greater than 1 shows a greater fitness for NewHG *pbp4*, and a value less than 1 a greater relative fitness for NewHG [46]. These values were plotted for each mouse organ (Fig 3E–3J). In no organ did the parental strain predominate. On the contrary, in livers (p = 0.0105), right kidneys (p = 0.0327) and spleen (p = 0.0476) there was a significantly higher proportion of *pbp4*, compared to its parent. Thus, the presence of PBP4, that is responsible for PG crosslinking asserts a fitness cost *in vivo*, in particular in the liver. This matches the biochemical observation of decreased oligomeric PG *in vivo*. However, virulence factors can also be niche specific, as *pbp4* has been shown to have a positive role in bone infection [47]. The decrease in crosslinking associated with loss of *pbp4* (with a higher proportion of monomers to oligomers) could also result in a greater activation of the innate immune system via induction of NOD2 [48], or NLRP3 activation [29].

### The role of the PG hydrolase SagB in infection

In the systemic zebrafish model of infection, a *sagB* mutation resulted in significant attenuation in both the NewHG and SH1000 strain backgrounds (S3A and S3B Fig). In this model bacteria are phagocytosed after infection, where subsequent emergence of bacteria leads to clonal lesion formation [49,50]. This model allows pathogen population dynamics to be determined to assess at what stage *sagB* is attenuated. At specified time points during ongoing infection, five infected living zebrafish larvae (and all dead) were collected and homogenised, after bacterial CFU was determined. For NewHG and SH1000 controls, with an infectious dose that kills approximately 50% of larvae, the bacterial numbers either remain similar to the inoculum or increase up to approximately 10^6^ CFU with concomitant death (S3C and S3E Fig). The otherwise isogenic *sagB* strains show similar kinetics but with less hosts succumbing and increased bacterial clearance (S3D and S3F Fig).

**Fig 4:**
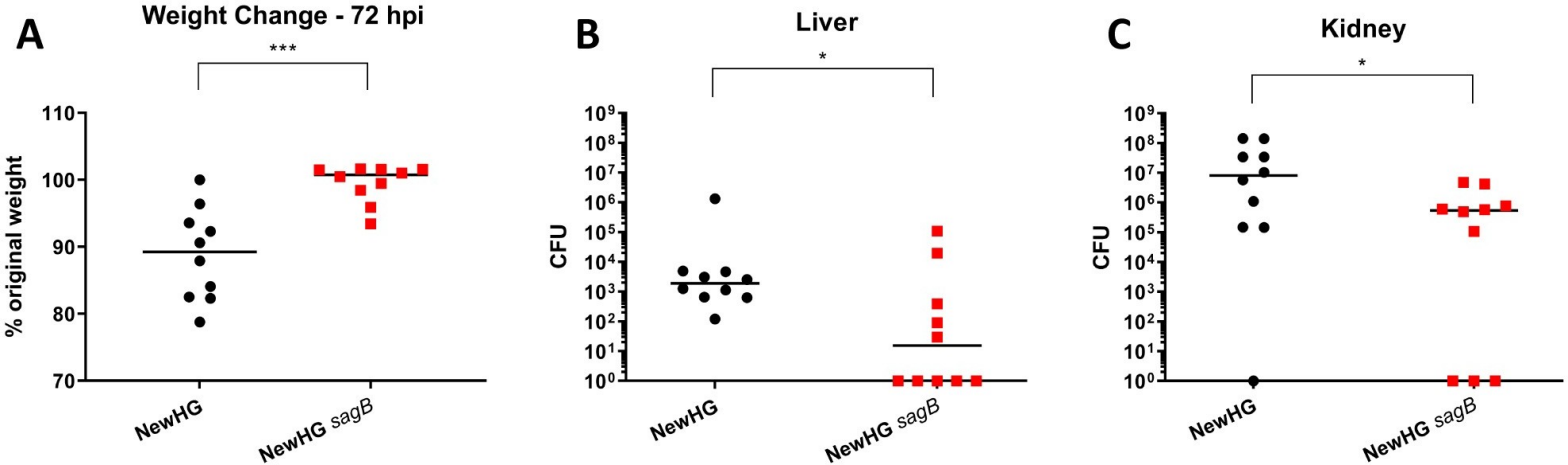
The role of SagB in *S*. *aureus* virulence. Approximately 1 x 10^7^ CFU of *S*. *aureus* NewHG *kan*^*R*^ (SJF 3680) or NewHG *sagB*::*kan* (SJF 4912) were injected intravenously into mice (n = 10). **(A)** Weight loss 72 hpi (p = 0.0005), **(B)** liver CFU (p = 0.0178) and **(C)** kidney CFU (p = 0.0368). Groups were compared using a Mann-Whitney U test (NewHG *kan*^*R*^–black circles, NewHG *sagB* red squares).

The attenuation of NewHG *sagB* was further investigated in the murine sepsis model of infection. Mice infected with NewHG *sagB* show significantly less weight loss that those infected with the parental strain (Fig 4A, p = 0.0005) and less *S*. *aureus* was recovered from the livers (Fig 4B, p = 0.0178) and kidneys (Fig 4C, p = 0.0178). This suggests that in murine infection, loss of SagB reduced bacterial fitness.

### The role of PG metabolism in murine macrophage interaction

**Fig 5:**
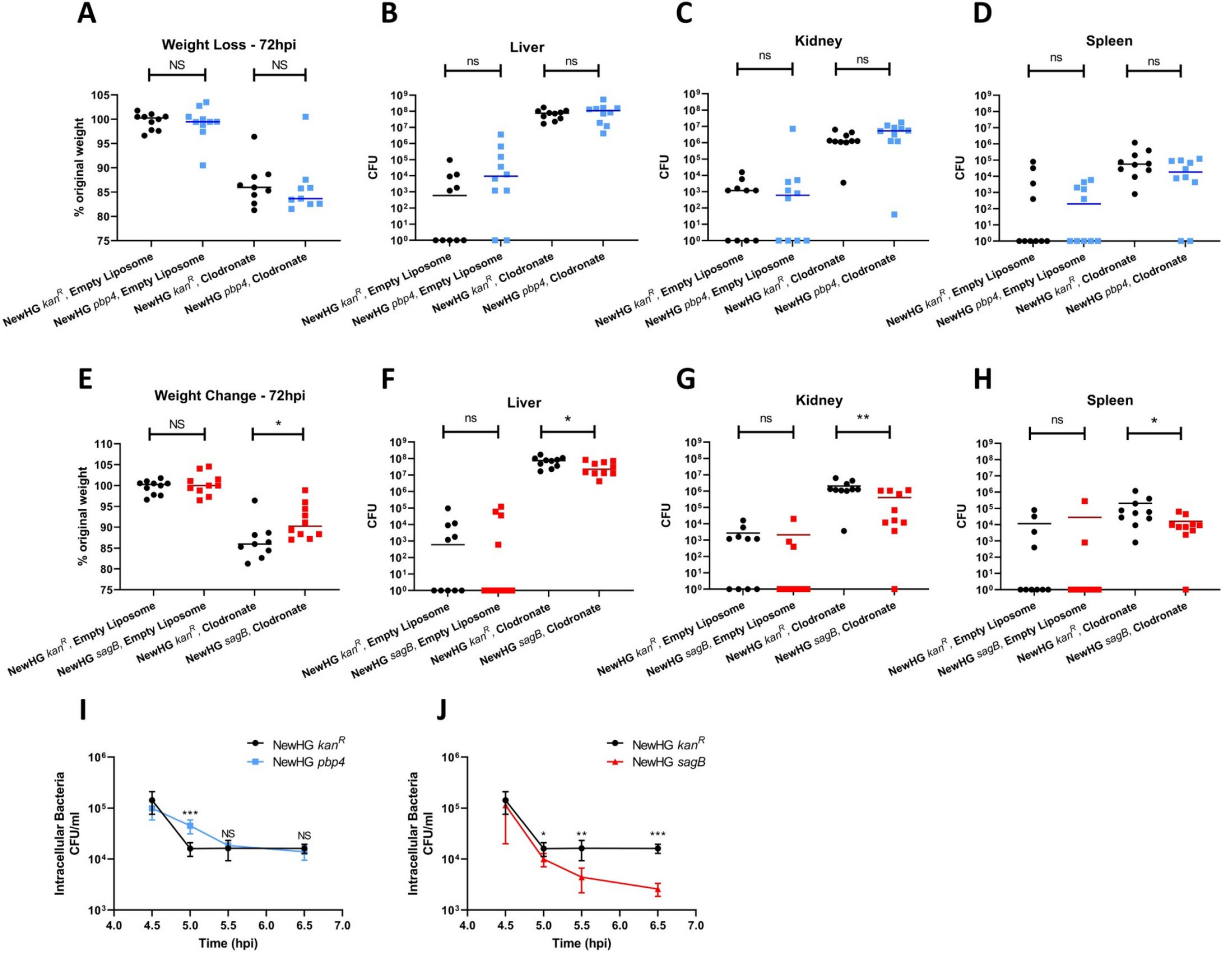
The role of peptidoglycan metabolism components and macrophages in *S*. *aureus* host-pathogen interactions. Mice (n = 10) were injected with approximately 1 x 10^5^ CFU of NewHG *kan*^*R*^ (SJF 3680), NewHG *pbp4*::*ery* (SJF 5103) or NewHG *sagB*::*kan* (SJF 4912) 24 hours post treatment with either empty liposomes or clodronate containing liposomes. 72 hpi, mice were sacrificed and the weight change (**A, E,** * p = 0.0254) and liver (**B, F,** * p = 0.0243), kidney (**C, G,** ** p = 0.0030) and spleen (**D, H,** * p = 0.0196) CFU were determined. (NewHG *kan*^*R*^–black circles, NewHG *pbp4*::*ery—*blue squares, NewHG *sagB*::*kan*- red squares). One mouse in the NewHG *kan*^*R*^ clodronate treated group and the NewHG *pbp4* clodronate treated groups was culled at 48 hpi due to reaching severity limits and have been excluded from analysis. MDMs were infected with *S*. *aureus* NewHG *kan*^*R*^ (SJF 3680, black bars), **(I)** NewHG *pbp4*::*ery* (SJF 5103, blue bars) or **(J)** or NewHG *sagB*::*kan* (SJF 4912, red bars) at a MOI of 5 (1 x 10^6^ CFU) for 4 hours before being treated with gentamycin for 0.5 hours to kill extracellular bacteria. MDMs were lysed at specific time points and intracellular bacterial numbers were determined. Paired two-tailed t-tests were used to compare between the strain CFU at subsequent time points. (For **(I)** *** p = 0.0008 and for **(J)** * p = 0.0250, ** p = 0.0075, *** p = 0.0003) Error bars show ±SD. (n = 3, each consisting of 2 intra-assay repeats).

In the murine sepsis model, Kupffer cells are primary gatekeepers to capture and control *S*. *aureus* circulating in the blood [4,51]. To determine if the roles in pathogenesis for PBP4 and SagB are associated with *in vivo* macrophage interactions, clodronate [52] was used to ablate these phagocytes. Clodronate treated mice are more susceptible to infection, so a lower inoculum of 1 x 10^5^ CFU NewHG, *pbp4* or *sagB* was used, with mice being injected with empty (control) or clodronate containing liposomes (Fig 5). With this low inoculum the vehicle liposome mice were able to control infection, maintain weight, and had residual bacterial numbers in organs, with no significant differences between the parent and mutants. However, clodronate led to a severe infection with the parental NewHG strain, with substantial weight loss and high CFU in both liver and kidneys. Loss of PBP4 had no effect on virulence (Fig 5A–5D) in the clodronate treated mice, alluding to a role for macrophages in the observed increase in recovery of *pbp4* in untreated mice (Fig 3B). Conversely the *sagB* mutant was still attenuated as measured by weight loss (Fig 5E, p = 0.0254) and CFU in the liver (Fig 5F, p = 0.0243), kidneys (Fig 5G, p = 0.0030) and spleen (Fig 5H, p = 0.0196). Therefore, macrophages can at most only be part of the reason why *sagB* is attenuated.

### The role of PG metabolism in human macrophage interaction

To further determine the role of PG metabolic components in host interaction, human macrophages were used. Monocyte derived macrophages (MDM, 2 x 10^5^) were infected with 1 x 10^6^ CFU NewHG *pbp4* or NewHG (multiplicity of infection (MOI) of 5) for 4 hours. At 4.5 hpi there is no significant difference in the NewHG *kan*^*R*^ or NewHG *pbp4* CFU recovered from lysed macrophages (Fig 5I). However, at 5 hpi, significantly more NewHG *pbp4* are recovered from macrophages than NewHG (p = 0.0008). At 5.5 and 6.5 hpi no differences were observed in the recovery of internalised NewHG or NewHG *pbp4*. This suggests that NewHG *pbp4* are more able to survive initial macrophage killing but succumb later. However, as CFU outcome is a product of both bacterial proliferation and survival, it is possible that the *pbp4* mutant has an initial intracellular growth advantage over the wildtype strain. The survival of NewHG *pbp4* was also investigated in neutrophils, where no difference in uptake (S2S Fig) or CFU recovered from neutrophils (S2T Fig) was found compared to the parental strain.

The role of SagB in MDM interactions was also tested as above (Fig 5J). The intracellular NewHG *sagB* CFU recovered at 5 (p = 0.0250), 5.5 (p = 0.0075) and 6 hpi (p = 0.0003) are all significantly lower than those recovered for NewHG, demonstrating NewHG *sagB* is less able to survive within host macrophages. However, reduced intracellular proliferation could also be the cause of fewer CFUs being recovered for the NewHG *sagB*.

### Role of PG metabolism components in augmentation of pathogenesis

Recently, the phenomenon of augmentation of infection has been described, whereby co-inoculation of *S*. *aureus* with commensal organisms, or cell wall PG, results in the ability of a much lower inoculum to cause disease [18]. Augmentation occurs within Kupffer cells resulting in increased *S*. *aureus* survival and subsequent proliferation [18]. Given the ability of PG to elicit augmentation, we determined the role of the metabolic components. Purified PG could be contaminated with lipoproteins, which are known to be immunobiologically active [53]. However, PG isolated and purified from an *lgt* mutant (which has no lipoprotein) has previously been shown to augment infection [18]. Mice were injected with a low dose (1 x 10^6^ CFU) NewHG or NewHG *pbp4*, with or without the addition of 250 μg purified *S*. *aureus* NewHG PG. Augmentation of NewHG resulted in more weight loss (p = 0.0079) and increased CFU in the liver (p = 0.0079) for both NewHG and its *pbp4* derivative (S4 Fig). There was a significant increase in CFU recovered from NewHG *pbp4* infected livers, compared to wildtype infected, but this was lost upon augmentation (S4 Fig; p = 0.0397 and 0.2857, respectively).

Both NewHG and its *sagB* derivative demonstrated augmentation in terms of weight loss (S4 Fig; p = 0.0079 and 0.0159, respectively), however, only NewHG liver CFUs increased when augmented with PG (p = 0.0079 and 0.0952, respectively). These data suggest that neither the activities of SagB or PBP4 are required for augmentation to occur.

### The combined role of PG synthases and hydrolases in *S*. *aureus* virulence

**Fig 6:**
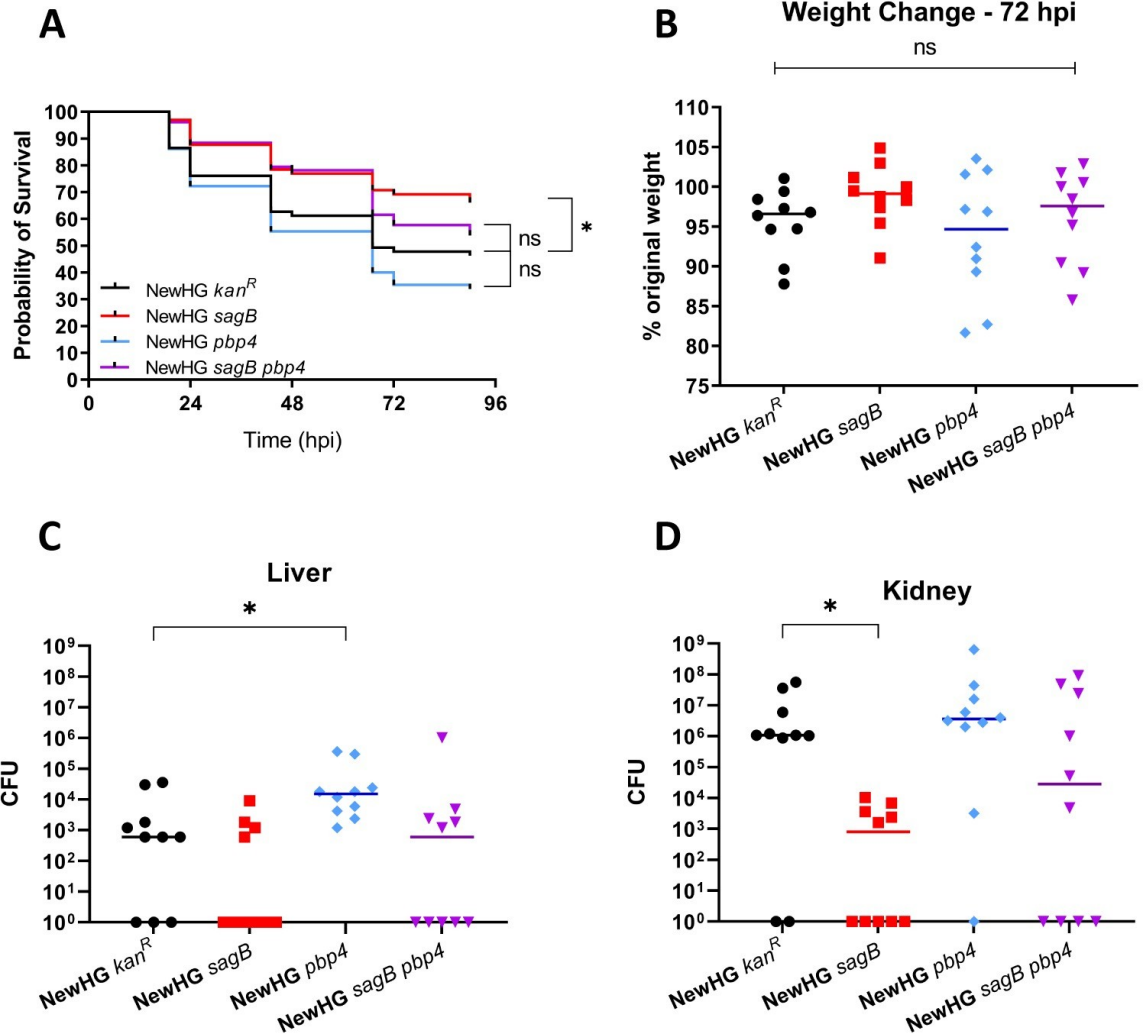
Analysis of growth and virulence of NewHG *sagB pbp4*. **(A)** Approximately 1500 CFU of bacteria (mutant or wildtype) was injected into the circulation valley of LWT zebrafish embryos around 30 hpf. A survival curve was produced comparing the virulence of parental NewHG (SJF 3663, black line) to: NewHG *sagB*::*kan* (SJF 4912, red line), NewHG *pbp4*::*ery* (SJF 5103, blue line) and NewHG *sagB*::*kan pbp4*::*ery* (SJF 5147, purple line). (3 repeats, n>20, * p = 0.0157). Mice (n = 10) were injected intravenously with approximately 1x10^7^ CFU *S*. *aureus* NewHG *kan*^*R*^ (WT, SJF 3680), NewHG *sagB*::*kan* (SJF 4912), NewHG *pbp4*::*ery* (SJF 5103) or NewHG *sagB*::*kan pbp4*::*ery* (SJF 5147). **(B)** Weight loss 72 hpi and CFUs recovered from **(C)** livers (* p = 0.0119) and **(D)** kidneys (* p = 0.0055). Groups were compared using a Mann-Whitney U test (NewHG *kan*^*R*^–black circles, NewHG *sagB*::*kan–*red squares, NewHG *pbp4*::*ery* blue diamonds, NewHG *sagB*::*kan pbp4*::*ery–*purple triangles).

Loss of PBP4 resulted in increased *S*. *aureus* fitness in the livers of mice, while a loss of SagB resulted in decreased virulence. The combined roles of SagB and PBP4 was therefore tested. In the zebrafish model of infection, *sagB* was attenuated compared to NewHG (p = 0.0157), whereas *pbp4* and *pbp4 sagB* were not (p = 0.2002 and 0.1602, respectively). NewHG and otherwise isogenic *pbp4*, *sagB* and *pbp4 sagB* were compared in the murine sepsis model (Fig 6). Loss of PBP4 led to increased CFU in the liver (p = 0.0119) and *sagB* decreased kidney numbers (p = 0.0053), compared to NewHG as expected. NewHG *pbp4 sagB* did not show a significant difference in terms of weight loss or organ CFU compared to NewHG (Figs 6 and S5). Using Fisher’s test to compare the ratios of infected and non-infected livers (Fig 6C) between strains, it was found that there were significant differences between the ratios of uninfected and infected livers between *sagB* and *pbp4* (p = 0.0108) and *pbp4* and the *pbp4 sagB* mutants (p = 0.0325). No significant differences were found between groups in the kidneys using this analysis. This suggests that the combination of the two mutations result in a compensatory effect on virulence.

### The role of the glucosaminidases in virulence

**Fig 7:**
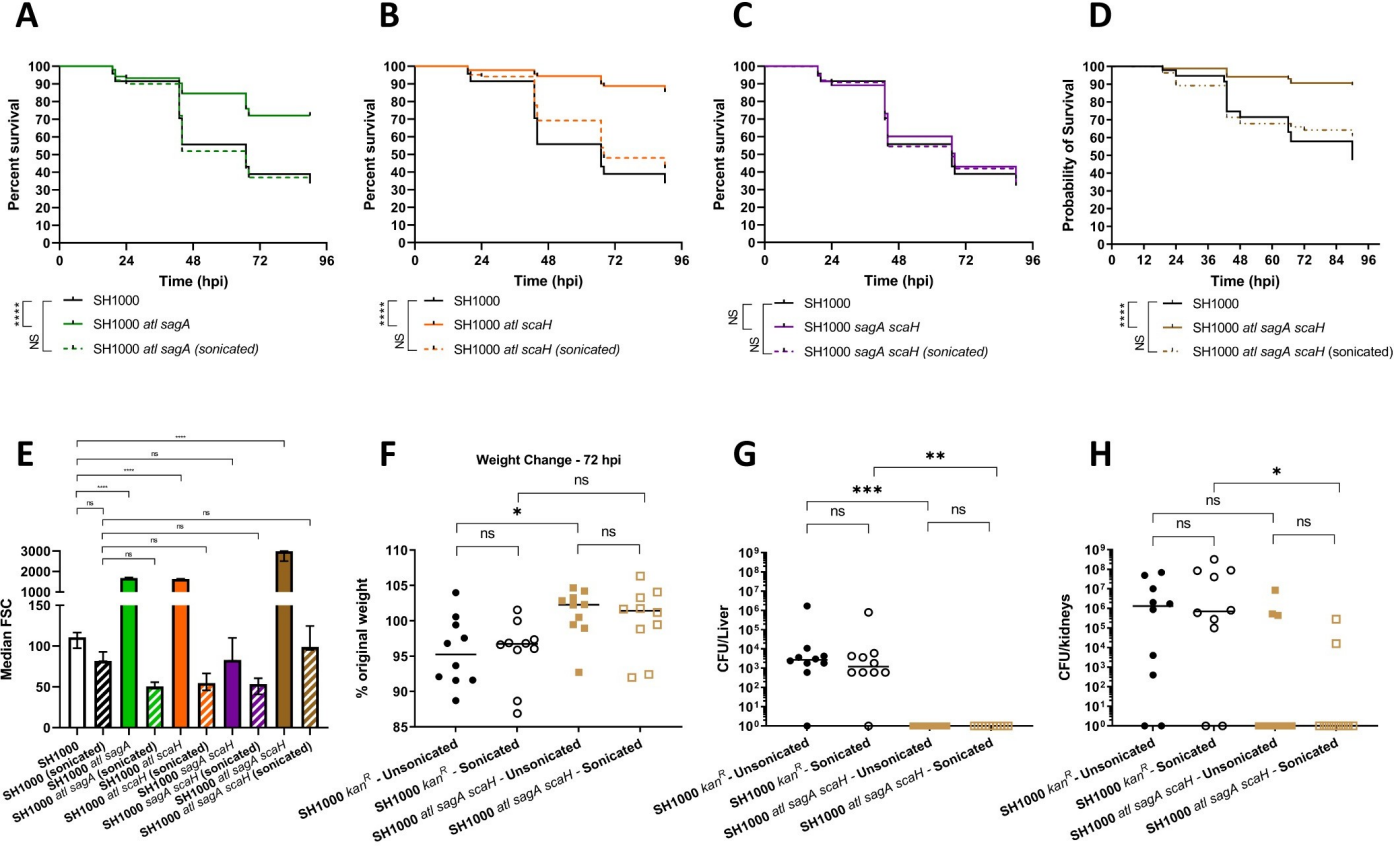
Role of putative glucosaminidases in *S*. *aureus* growth and virulence. Survival curves comparing the virulence of 1300 CFU parental NewHG (SJF 3663, black lines) to **(A)** 400 particles unsonicated SH1000 *atl sagA* (SJF 5261, solid green line) consisting of 1300 bacteria (when sonicated) or approximately 1300 CFU of sonicated SH1000 *atl sagA* (broken green line). **(B)** 300 particles of unsonicated SH1000 *atl scaH* (SJF 5262, solid orange line) consisting of 1300 bacteria (after sonication) or 1300 CFU sonicated SH1000 *atl scaH* (broken orange line). **(C)** 1300 CFU unsonicated SH1000 *sagA scaH* (SJF 5217, solid purple line, sonication did not change CFU) or 1300 CFU sonicated SH1000 *sagA scaH*. **(D)** 100 particles of unsonicated SH1000 *atl sagA scaH* (consisting of 1500 bacteria, solid brown line), 1500 CFU of sonicated SH1000 *atl sagA scaH* (broken brown line). (A-D each consist of 3 repeats, n>20, **** p < 0.0001). **(E)** Comparison of the median FSC light values of parental SH1000 (unsonicated white bar, sonicated black and white bar), SH1000 *atl sagA* (unsonicated green bar, sonicated green and white bar), SH1000 *atl scaH* (unsonicated orange bar, sonicated orange and white bar) SH1000 *sagA scaH* (unsonicated purple bar, sonicated purple and white bar) and SH1000 *atl sagA scaH* (unsonicated brown bar, sonicated brown and purple bar). (n = 3, **** p < 0.0001). Error bars show ±SD, FSC values were compared using a one-way ANOVA with Dunnet’s multiple comparison test. **(F-G)** *S*. *aureus* SH1000 *kan*^*R*^ (SJF 3674) or SH1000 *atl sagA scaH* (SJF 4611) were injected intravenously into mice (n = 10). Approximately 1 x 10^7^ bacteria were injected in unsonicated and sonicated groups. 1 x 10^7^ bacteria in 5 x 10^6^ particles of unsonicated SH1000 *atl sagA scaH* was injected (sonication had no effect on SH1000 *kan*^*R*^). **(F)** Weight loss 72 hpi (* p = 0.0314), **(G)** liver CFU (** p = 0.0014, *** p = 0.0003) and **(H)** kidney CFU (** p = 0.0096) were determined. Groups were compared using a Kruskal-Wallis test with multiple comparisons (Unsonicated SH1000 *kan*^*R*^–black circles, sonicated SH1000 *kan*^*R*^–open black circles, unsonicated SH1000 *atl sagA scaH—*brown squares and sonicated SH1000 *atl sagA scaH–*open brown squares). Sonication was for 20 seconds at an amplitude of 5 microns when required.

SagB is a member of a family of 4 putative glucosaminidases (SagB, Atl, SagA, ScaH) encoded on the *S*. *aureus* chromosome [34,35]. As a *sagB* mutant was found to be attenuated in both the zebrafish and murine models of infection, it was hypothesised that other putative glucosaminidases of *S*. *aureus* (Atl, SagA and ScaH) may also play a role in infection. The SH1000 background was used for this part of the study as the range of mutants were available and had been previously characterised in terms of biochemistry and physiology [35]. For the single mutants (*atl*, *sagA*, *scaH*) no differences in pathogenesis were observed in the zebrafish *S*. *aureus* infection (S6 Fig). It is known that Atl is required for cell separation [35,36,54] and so a sonicated inoculum was used to ensure an accurate CFU was administered. The loss of SagA or ScaH did not affect cell separation alone (S6 Fig). Given the functional redundancy previously observed for PG hydrolases a series of double and a triple mutant were tested. Multiple mutant strains containing the *atl* mutation all showed a clustering phenotype during growth (S6 Fig) and by flow cytometry (Fig 7E), which could be reversed by sonication. To test virulence of the strains, the equivalent of 1300 bacteria were injected into zebrafish embryos (Fig 7). 400 particles of SH1000 *atl sagA*, 300 particles of SH1000 *atl scaH* or 1300 particles SH1000 *sagA scaH*, all consisting of approximately 1300 bacteria, were injected into embryos and compared to sonicated particles and SH1000. Embryos injected with unsonicated SH1000 *atl sagA* or *atl scaH* showed significantly greater survival than those injected with SH1000 (both p < 0.0001) and full virulence of the inoculum was restored by sonication prior to injection. Both untreated and sonicated SH1000 *sagA scaH* showed no difference in virulence compared to SH1000 in the zebrafish model of infection (Fig 7C). The triple SH1000 *atl sagA scaH* mutant also has a cell separation defect and attenuated virulence that can be ameliorated by sonication (Figs 7 and S7). Thus, the number or size of cell particles inoculated has a profound effect on infection outcome.

The virulence of SH1000 *atl sagA scaH* was further tested in the mouse sepsis model of infection. SH1000 *kan*^*R*^ was used as the control [55] to allow selection of *S*. *aureus* from organ homogenates. Mice were injected with unsonicated or sonicated SH1000 or SH1000 *atl sagA scaH*, of known particle and CFU number. Mice were injected with 1.6 x 10^7^ CFU in 1.5 x 10^7^ particles of unsonicated SH1000, or 1.6 x 10^7^ CFU of sonicated bacteria. 1.4 x 10^7^ bacteria in 5.6 x 10^6^ particles of unsonicated SH1000 *atl sagA scaH* or 1.5 x 10^7^ CFU of sonicated bacteria were used. As opposed to the zebrafish model, using a sonicated versus an untreated inoculum did not affect the outcome in terms of weight loss or organ CFU for SH1000 or *atl sagA scaH*. However, the unsonicated triple mutant is attenuated in terms of weight loss (p = 0.0314) and liver CFU (p = 0.0014) compared to SH1000 (Fig 7). Sonication of organs after harvesting, to disrupt potential bacterial clusters, did not significantly alter organ CFU (S7 Fig). Thus, SH1000 *atl sagA scaH* is attenuated in the murine sepsis model and, unlike in the zebrafish model, sonication to disperse clusters has no impact on virulence in this system.

To determine if the alterations in PG structure and cell clustering effect the augmentation of *S*. *aureus* infection by PG the pathogenesis of *atl sagA scaH* was determined in the presence of PG from SH1000 or *atl sagA scaH* (S7 Fig). As *atl sagA scaH* is attenuated, an inoculum of 1 x 10^7^ CFU of sonicated SH1000 *atl sagA scaH* was used for augmentation. Experiments to examine augmentation were carried out using 250 μg of PG from SH1000 or SH1000 *atl sagA scaH*. Successful augmentation occurred with both types of PG resulting in significant weight loss and increase in CFU in the livers and kidneys (S7 Fig; all p < 0.05). For spleens, lungs and hearts, no significant difference between recovered CFU could be seen between groups (S7 Fig). This demonstrates that PG of diverse origins can augment pathogenesis [18] and that produced from *atl sagA scaH* was still capable of enhancing infection.

## Discussion

We report the first PG structure from bacteria obtained during an infection. The muropeptide profile, paired with TEM analysis, has helped to elucidate the structure of the *S*. *aureus* cell wall during disease. Defining host-pathogen interactions increases understanding of the infection process, thereby providing new information for developing treatment, especially important in the ever-approaching post-antibiotic era. Previous research has determined that the growth phase of *S*. *aureus* influences the immune response of infected dendritic cells, which is also influenced by exogenous PG [56]. Knowing the structure and composition of PG during an infection could therefore provide more detail on *S*. *aureus* interactions with the host immune system.

We show that *S*. *aureus* has reduced PG crosslinking during an infection than when cultured *in vitro*. Many surface proteins are required in abscess formation [57,58], which are bound to the terminal glycine in the pentapeptide bridge [59]. The observed reduction in crosslinking in PG recovered from infected kidneys could be due to glycine bridges being used to anchor cell surface proteins required for abscess formation, at low growth rates, permitting survival and pathogenesis in the host, rather than crosslinking PG. A reduced muropeptide diversity was presented in this study (Fig 2 and S4 Table), suggesting that PG from *S*. *aureus* has less complexity and crosslinking during an infection. It has been hypothesised that in *S*. *epidermidis* PG synthesis is reduced when exposed to human platelets, creating a less complex PG structure [41]. This allows energy to be used in other processes, allowing proliferation of bacteria in a harsh, restrictive environment such as infection [41]. *Salmonella enterica* serovar Typhimurium cultured within human epithelial cells possesses a unique PG structure, with a reduction in glycine containing muropeptides and a decrease in crosslinking [60]. The methodology of our study did not allow the detection of O-acetylated muropeptides, which may change in abundance during infection [13,14].

During infection, *S*. *aureus* is likely to be in a state resembling stationary phase (Fig 2). The PG structure of *S*. *aureus* is influenced by nutrient availability during growth, and this is the likely cause for the reduced PG crosslinking. As cells reach stationary phase glycine depletes as it is used for protein and cell wall production [11]. This coincides with a thickening of the cell wall and the production of irregular septa with an increased number of non-crosslinked side chains [11]. These changes occur in the newly synthesised PG rather than a modification to existing PG [11]. In a *S*. *aureus* biofilm, non-crosslinked pentaglycine bridges are used as attachment sites for proteins which are less abundant in planktonic culture [61]. *S*. *aureus* cells propagate in diverse environments and in the host with each organ provides a different nutrient and immunological repertoire [62], which could result in differing PG structures during an infection. Bacteria grow rapidly once in a niche, and quickly run out of resources, with the abscesses within the kidney being self-limiting [4]. While within macrophages, *S*. *aureus* relies on nutrients to be delivered from the extracellular milieu of the host [63]. A reduction in cell size has previously been found in viable *S*. *aureus* cells undergoing starvation *in vitro* [43], suggesting a change in metabolism and growth phase may be the reason for the reduction in cell size and increased cell wall thickness observed in our study. *S*. *aureus* has also been observed to have thicker cell wall material when intracellular within human or mouse osteoblasts [64].

The role of PG metabolism in virulence was also investigated within our study due to the observed changes in cell wall structure (thicker cell walls) and muropeptide profile (less crosslinked species) in *S*. *aureus* recovered from an infection. A *pbp4* mutant (with reduced PG crosslinking) demonstrates greater colonisation of mouse livers in the murine sepsis model of (Fig 3). This complements previous findings that a *pbp4* mutant causes larger skin lesions in infected mice, which was suggested to be caused by an increase in production of IL-1β [29]. Similarly, inactivation of the ClpXP protease, which results in thicker cell walls and increased peptidoglycan cross-linking [65], resulted in a less expression of IL-1β in mouse bone marrow-derived macrophages [29]. In a murine model for implant-associated osteomyelitis it was found that the loss of *pbp4* resulted in no change in bacterial loads and attenuation in regards to bone loss [47]. Our study shows an increase in liver CFUs but with no change in weight loss in mice, suggesting no overall increase in virulence in the *pbp4* mutant. Liver phagocytes take up the majority of free *S*. *aureus* in the blood during a murine infection [4], with bacteria being taken up by Kupffer cells [51]. This suggests that a change in IL-1β production may occur during infection dependent on the crosslinking of PG, with reduced PG crosslinking resulting in greater colonisation in the liver. In a *S*. *aureus oatA* mutant which does not have O-acetylated PG, the PG can be degraded by lysozyme which results in IL-1β induction [66], suggesting that PG crosslinking and degradability is important in pathogenesis and the host response. A hypothesised change in PG crosslinking may also facilitate colonisation of livers through resistance to macrophage killing (Fig 5I), which could also be explained by changed induction of macrophage antimicrobial products, or a reduction in the secretion of proinflammatory cytokines [29].

The observed *in vivo* increase *in S*. *aureus* cell wall thickness and proportion of cells which fail to separate after septation (Fig 1) suggests an alteration in PG hydrolase activity compared to *in vitro* growing cells. The loss of the major glucosaminidase SagB has recently been shown to lead to thinner cell walls [40] and thus SagB was hypothesised to be involved in the increased cell wall thickness and have a role in disease. Investigation into the glucosaminidases which revealed different roles for the members of this class of enzymes. A loss of SagB alone results in attenuation, whereas a combination of the other three (Atl and SagA or ScaH) is required for attenuation. Understanding the role of PG hydrolases allowed us to discern the reasons for the triple (Atl, SagA, ScaH) attenuation as these function in cell separation. It has previously been shown in *Enterococcus faecalis* that loss of AtlA activity results in long cell chains and attenuation in the zebrafish model of infection, reversible upon chain disruption [67]. Our study has shown that in *S*. *aureus* the loss of Atl (the major PG hydrolase) activity alone is not enough to cause attenuation in a zebrafish model of infection (S6C Fig). SagA and/or ScaH activity must also be inactive to result in attenuation that is reversible on cluster disruption (Fig 7). However, we have also shown that in a murine model of infection cluster, disruption does not restore virulence, suggesting that inhibiting *S*. *aureus* cell cleavage could potentially be a promising route for new clinical treatments. This disparity could be due to there being a greater number of immune bottlenecks in a murine infection compared to zebrafish [4], resulting in clusters reforming at different points within the host. The size of bacterial cells and particles has previously been shown to be important during infection. Uropathogenic *E*. *coli* form filaments by inhibiting septation, resulting in resistance to phagocytosis and increased survival [68,69]. In *Enterococcus faecalis* loss of AtlA (PG hydrolase dedicated to septum cleavage) results in long chains of cells, which are attenuated within zebrafish due to their susceptibility to phagocytosis [67]. Similarly in *Streptococcus pneumoniae* infection, increasing chain length increases the pathogen’s susceptibility to phagocytosis due to increasing complement deposition [70]. *In vitro* experiments in alveolar macrophages using polystyrene particles of different shapes and sizes has shown that particle size influences the completion of phagocytosis, even when the particle has a greater volume than the phagocyte [71]. Particle shape and the point of contact between the particle and macrophage was found to be more important for phagocytosis initiation [71]. The formation of clumps reduces the ability of *S*. *aureus* to disseminate during an infection, as similarly proposed for *E*. *faecalis* [67]. The reduction in dissemination and multiple bacteria forming single particles potentially limits the number of infectious events being initiated. It has been proposed that *S*. *aureus* infection dynamics requires the interaction with phagocytes for infection and dissemination to occur [4]. Therefore, the lack of dissemination from clustering results in fewer phagocytosis events resulting in *S*. *aureus* infection establishing, and therefore less chance of causing mortality in the zebrafish model of infection.

While macrophages play a role in the attenuation of *sagB* mutants, it is not the only cause of attenuation (Fig 5). The inactivation of *sagB* has been shown to result in a slight growth defect and longer glycan chains in PG, resulting in a stiffer cell wall which impairs cells in their ability to increase in size post division and adopt the correct mature shape [35]. The PG architecture of a *sagB* mutant has recently been shown to take longer to reach maturity [40], and this could change interactions with the host [56], resulting in attenuation. The change in architecture could also lead to a change in the surface display of proteins due to a change in the ability to export proteins across the cell wall. It has already been observed that *sagB* mutants have an aberrant protein secretion phenotype [34], which is likely also a cause of attenuation. Secreted proteins, including those important to virulence, such as: staphylococcal complement inhibitor, staphylococcal superantigen-like protein 1 (SSL1), SSL7, SSL11 and Coa were found in reduced concentrations in the culture medium when compared to wildtype [34]. The secretion defect was also found to include the increased secretion of 251 proteins, 90.5% (227 proteins) of which were predicted to be cytoplasmic [34]. Also previously, disruption of the pentaglycine crosslink in PG in a *Staphylococcus carnosus femB* mutant has been shown to result in the release of cytoplasmic proteins and lipoproteins [72]. Thus the increased secretion of cytoplasmic proteins would help to explain both the reduced growth rate *in vitro* and virulence, as intracellular proteins erroneously excreted to the extracellular environment cannot perform their function. Altered release of proteins due to loss of *pbp4* or *sagB*, could account for virulence changes as cytoplasmic proteins such as aldolase and GAPDH have been shown to contribute to virulence in *S*. *aureus* [73]. *S*. *aureus sagB* mutants have been reported to not show an altered autolysis phenotype [34], so lysis is not a cause of attenuation.

PG is known to be an important factor in the initiation of infection, being able to augment *S*. *aureus* resulting in an altered disease, with an increased liver colonisation in mice [4,18]. A reduction in PG crosslinking could result in an increased release of PG turnover products within the phagosome, augmenting infection [18]. Conversely, the loss of PG hydrolase activity, such as with SagB, would result in the release of fewer PG turnover products within a phagosome, reducing the augmentation potential of the bacteria. It is also possible that PG turnover products with different chain lengths and crosslinking may have inherently different augmenting capabilities, resulting in a change in immune outcome. PG may interact with antimicrobials produced by phagocytes [74], neutralising them. Different PG structures could therefore have different capabilities for antimicrobial neutralisation, and therefore different augmenting capabilities.

Our work paves the way for a fuller understanding of the importance of PG structure in *S*. *aureus* disease and assists the discovery of new ways to control this insidious pathogen.

## Materials and methods

### Ethics statement

Murine work was carried out according to UK law in the Animals (Scientific Procedures) Act 1986, under Project License P3BFD6DB9 (*Staphylococcus aureus* and other pathogens, pathogenesis to therapy, University of Sheffield Review Board).

### *S*. *aureus* strains, growth conditions and growth curves

The *S*. *aureus* strains used in this study are described in S1 Table. *S*. *aureus* transposon mutants were obtained from the NARSA library (http://www.narsa.net). The transposons from selected strains were transferred into other *S*. *aureus* strains as required by Φ11 transduction. Genomic DNA was isolated using Qiagen DNeasy Blood and Tissue kit (Cat no. 69506) in accordance with manufacturer’s instructions. Prior to DNA extraction 5–10 μl of 5 mg/ml lysostaphin was added to resuspended *S*. *aureus* cells to facilitate lysis. Successful transduction was confirmed using PCR with the appropriate oligonucleotides listed in S2 Table.

Standard conditions for growth used solid or liquid tryptic soy broth (TSB) supplemented with erythromycin (5 μg/ml) plus lincomycin (25 μg/ml), kanamycin (50 μg/ml) plus neomycin (25 μg/ml), tetracycline (5 μg/ml) or spectinomycin (100 μg/ml). Bacteria were cultured in a water bath at 37°C with shaking at 200 rpm to produce a culture at mid-exponential phase (OD_600_ 0.4–0.8) or stationary phase (OD_600_ = 8–10) cultures. Direct cell counts were performed to quantify viable bacterial numbers by serial dilution in sterile PBS. The number of colony forming units (CFU) were directly counted from the plates after incubation. Growth curves were performed in triplicate and growth was measured hourly by direct cell counts and/or OD_600_ measurements. Sonication was performed with a Soniprep 150 Plus bench-top ultrasonic disintegrator (MSE) with an exponential microprobe (tip diameter of 3 mm), with 400 μl of sample sonicated at an amplitude of 5 microns for 20 seconds on ice.

### Transmission electron microscopy (TEM)

After organ CFU had been determined 1 ml of organ homogenate was centrifuged to produce a pellet (16873 x g, 10 min, room temperature). For *in vitro* derived samples 1 ml of culture at the appropriate growth phase was centrifuged (16873 x g, 10 min, room temperature) to produce a pellet. The samples were then treated for TEM analysis. Briefly, samples were mixed with 2.5% (w/v) glutaraldehyde and left overnight at 4°C to fix. Glutaraldehyde was removed and samples were washed with PBS. Samples were mixed with 2% (w/v) aqueous osmium tetroxide for 2 hours (at room temperature) for secondary fixation. Excess osmium tetroxide was removed using two PBS washes. Samples were dehydrated by the addition of incremental concentrations of ethanol (75% v/v, 95% v/v and 100% (v/v) ethanol) for 15 min each before being removed and the higher concentration added. Samples were incubated twice with propylene oxide to complete dehydration. Samples were mixed with a 50% propylene oxide to 50% Epon resin mixture and left overnight at room temperature to allow infiltration of the sample. The resin was removed, and any remaining propylene oxide was removed by evaporation. Pure Epon resin was added to the sample, which was left for 4 hours, after which the resin was removed and replaced with fresh pure Epon resin for another 4 hours. Epon resin was removed and the samples were imbedded in fresh resin. Resin polymerisation was performed at 60°C for 48–72 hours.

80 nm thin sections of the samples were produced using an Ultracut E Ultramicrotome (Reichert-Jung) at room temperature. The thin sections were mounted onto 200-square mesh copper TEM grids (Agar Scientific) already treated with a 1.5% Pyroxylin (w/v, in amyl acetate) film. Mounted sections were stained in 3% (w/v) aqueous uranyl acetate for 30 min and washed with dH_2_O. Sections were then stained with Reynold’s lead citrate [75] for 5 min and washed with dH_2_O.

Sections were imaged using a FEI Tecnai T12 Spirit Transmission Electron Microscope operating at 80 kV. Images were recorded using a Gatan Orius SC1000B bottom mounted CCD camera. TEM images were analysed using Fiji software [76]. Cell area was calculated using adapted methodology [77]. Two perpendicular measurements were made along the cell, from the edge of the cell wall to the edge of the cell wall. The measured radii were then used to calculate the estimated cell area. For cell wall measurements, four equidistant cell wall measurements were made around the cell where the cell wall was clearly defined [78].

### Purification of PG

*S*. *aureus* cultures of the appropriate growth phase were centrifuged (16873 x g, 10 min, 4°C) to harvest cells and resuspended in Tris HCl pH 7.5 containing 2% SDS (w/v) and boiled for 10 min to kill cells and inactivate enzymes before being washed once with dH_2_O. Boiled cells were added to lysing matrix tubes containing 0.1 mm silica beads (Lysing Matrix B, MP Biomedicals). The cells were sheared 10 times at 6.0 m/s for 30 sec using an MP Biomedicals FastPrep 24 Homogeniser, with samples being kept on ice between cycles. Sacculi were separated from the lysing matrix by centrifuging for 30 sec at 100 x g and collecting the supernatant. Sacculi were harvested from the supernatant and resuspended in 4% (w/v) SDS and boiled for 30 mins, and then resuspended in 50 mM Tris-HCl pH 7.5 containing 3% SDS (w/v), 50 mM DTT, 1.25 mM EDTA and boiled for 30 mins. Remaining SDS was removed by washing the pellet six times in dH_2_O. Pellets were resuspended in 50 mM Tris HCl pH 7.5 containing 2 mg/ml pronase and incubated for 30 mins at 60°C. Wall teichoic acids and any other remaining cell wall polymers were removed by incubating the sacculi in 250 μl 48% (v/v) hydrofluoric acid (HF) at 4°C for 48 hours. The purified sacculi were washed in alternating 50mM Tris HCl pH 7.5 and dH_2_O until the pH was raised to at least 5.0, ending on a dH_2_O wash to remove Tris HCl residue. Purified sacculi were harvested by centrifugation and stored at -20°C until required.

To produce purified PG (murein) derived from murine infection, individual kidneys were homogenised in 2 ml PBS using a PreCellys 24-dual (Peqlab) homogeniser. Kidneys (from 10 mice) were pooled from a single mouse experiment that shared a starting inoculum. Insoluble material was recovered by centrifugation at 16873 x g (Avanti centrifuge, JA 25.50 rotor) and resuspended in Tris HCl (50mM, pH 7.5) containing 2% SDS (w/v) and boiled for 10 min to kill cells. Material was washed twice in dH_2_O, and the pellet resuspended in 10 ml 50 mM Tris HCl pH 7.5. The mixture was transferred to a Braun Homogeniser bottle containing 50 g of sterile, acid-washed glass beads and placed on ice. The contents of the bottle were disrupted using a Braun homogeniser (Braun, Germany). Each bottle was homogenised for 10 x 30 sec, with 5 min on ice in-between each homogenisation. The beads were separated from the biological material with a vacuum sintered glass filter, and the resulting filtrate centrifuged (16873 x g, 10 min, 4°C, Avanti centrifuge, JA 25.50 rotor) to recover the bacterial sacculi and residual kidney matter. The resulting pellet was then purified in the same manner as *in vitro* derived material above, using SDS, pronase and HF.

### PG digestion, RP-HPLC and mass spectrometry analysis of muropeptides

Purified PG (~2 mg) was resuspended in 90 μl MilliQ water and 10 μl 50 mM ammonium formate buffer (pH 4.8) was added, along with 20 μl cellosyl (kindly provided by Hoechst, Frankfurt, Germany). The digestion mixture was incubated overnight at 37°C, 900 rpm in a thermal shaker (Eppendorf). Cellosyl was inactivated at 100°C for 10 min, and samples were centrifuged at 16873 x g for 10 min, reserving the supernatant. The supernatant was dried using a speed vac vacuum concentrator (Thermofisher) and resuspended in 25 μl of MilliQ water and 25 μl of 0.5M Ammonium carbonate buffer (pH 9.0). Samples were reduced at room temperature for 30 min by the addition of tetra methyl ammonium borohydride (around 2 mg). Samples were acidified using 5% (v/v) formic acid to pH 4.0–4.5, then reduced in volume to around 30 μl using a speed vac. 15 μl of sample was injected onto the HPLC system (Agilent 1100, with an ACE3 C-18AQ column (1 × 150 mm). RP-HPLC conditions were as follows: buffer A (ultra-pure Milli-Q water with 0.1% (v/v) formic acid) to a maximum of 50% buffer B (Acetonitrile with 0.1% (v/v) formic acid) over 128 min at a flow rate of 0.05 ml/min. Muropeptides were detected by UV at 205 nm. MS analysis was performed using a modified protocol of previously published methods [42]. Muropeptides were analysed by infusion MS by directing RP-HPLC eluate to the ion source on an LTQ mass spectrometer (Thermo). The spray voltage was set at 3.6 kV and the transfer capillary temperature at 200°C. Mass spectra were collected over the range m/z = 300–2000 with MS/MS fragmentation spectra triggered for ion signals >5 x 10^3^ intensity. Analysis was performed using Xcalibur QualBrowser v2.0 (Thermo).

### Integration of identified muropeptide peaks

For all UV-absorbance peak muropeptide identification by mass spectrometry (MS), the ion of greatest abundance within a peak was used for peak designation. For monomeric and dimeric muropeptide species, MS/MS was used to confirm the identity of the most abundant observed ion. For muropeptide species with greater crosslinking (oligomers), the mass of the most abundant ion found by MS was used to identify the UV-absorbance peak. Relative quantities of muropeptides were calculated by the normalised percentage area under each peak for which the chemical structure had been determined.

### Zebrafish model of infection

Up to 5 days post fertilization (dpf) zebrafish are not protected under the Animals (Scientific Procedures) Act 1986. However, all work was carried out according to the stipulations set out in Project License PPL 40/3574. London wild-type (LWT) strains were used for all zebrafish experiments in this study. Adult zebrafish were maintained by staff at the University of Sheffield Bateson Centre Zebrafish Facility. Adult fish were kept in a continuous re-circulating closed system aquarium with a light/dark cycle of 14/10 hours at 28°C. LWT zebrafish embryos/larvae were kept in E3 medium at 28.5°C.

Zebrafish embryos were dechorionated manually at roughly 28 hpf, before being anaesthetised in 0.02% (w/v) 3-amino benzoic acid ester (tricaine, Sigma) 30 hpf and injected with 1 nl of bacterial suspension from a calibrated glass needle, as previously described [50]. Injections into the zebrafish embryo circulation valley were performed using, a pneumatic micropump (WPI, PV820), a micromanipulator (WPI) and a dissecting light microscope (Leica). After injection, embryos were placed individually into 96-well plates with 250 μl of E3 per well, and survival (indicated by presence of a heartbeat) was recorded up to 90 hpi.

To quantify bacterial growth within zebrafish, single larvae were collected with 100 μl E3 medium into 2 ml cap containers (Peqlab). Embryos were individually homogenised using a PreCellys 24-dual (Peqlab) and the resulting homogenates were serially diluted in sterile PBS and plated onto TSA as 10 μl spots. The limit of detection for this experiment was 10 CFU/embryo. Clearance was defined as being below the limit of detection.

### Murine sepsis model of infection

Murine work was carried out according to UK law in the Animals (Scientific Procedures) Act 1986, under Project License PPL 40/3699 and Project License P3BFD6DB9 (*Staphylococcus aureus* and other pathogens, pathogenesis to therapy).

Mouse injections were performed as previously described [4]. 6–8 weeks old female BALB/c mice (Charles River Laboratories, UK) were housed in designated animal facilities using standard husbandry protocols. 100 μl *S*. *aureus* was injected intravenously into the tail vein of 7-week-old mice. Inocula CFUs were quantified by serial dilution and plating onto TSA and incubating overnight at 37°C, before directly counting CFUs. Weights were recorded daily, and wellbeing checks were performed twice daily. Mice were monitored and euthanised at 72 hpi (unless otherwise stated in the experimental design) or if mice reached severity limits described in the protocol defined by the project license.

Individual mouse organs were homogenised in PBS (livers in 3 ml, other organs in 2 ml) using a PreCellys 24-dual (Peqlab) homogeniser. CFU per organ was determined by plating 10 μl spots of serially diluted homogenate onto TSA for bacterial number determination. Statistical significance between groups was determined using a Mann- Whitney two-tailed test or a one way-ANOVA.

Purified PG required for injections was centrifuged for 2 minutes at 16873 x g and then suspended in sterile endotoxin free PBS before being sonicated at an amplitude of 10 microns for 30 seconds. PG was injected at a dose of 250 μg per mouse at the same time as the bacteria.

Clodronate and empty control PBS liposomes (Liposoma research, The Netherlands, clodronateliposomes.com) were injected as per manufacturer’s instructions. Clodronate and control liposomes were injected at a dose of 100 μL of suspension per 10 grams (stock 5 mg clodronate / mL) intravenously 24 hours before infection. Mice were then infected with 1 x 10^5^ CFU of bacteria.

Relative fitness experiments used a modified version of an existing protocol [46]. 20 mice were injected with a 1:1 ratio of each strain (totalling 1 x 10^7^ CFU) and culled after 72 hpi. Organs were individually collected and plated onto TSA containing antibiotics to allow the counting of the number of each strain within each homogenate. Relative fitness was calculated using the equation *w* = x_2_(1-x_1_) /x_1_(1-*x*_2_) (where w = relative fitness, x_1_ = starting mutant proportion and x_2_ = ending mutant proportion) [46]. Relative fitness within each organ was then analysed using a one sample Wilcoxon signed rank test, comparing the results to a theoretical median of 1, testing if strains deviate from equal fitness.

Clonality experiments were performed as previously described [4]. Strains carrying kanamycin or tetracycline resistance were used in this study to allow for selection. These resistances have previously shown to have no impact upon *S*. *aureus* virulence [55]. The resistance cassettes were transferred by Φ11 transduction into NewHG *pbp4* following the existing methodology [4,55], producing NewHG *pbp4 kan*^*R*^ (SJF 5136) and NewHG *pbp4 tet*^*R*^ (SJF 5135). Mice were infected with either wildtype marked strains in a 1:1 ratio or with the NewHG *pbp4* marked strains in a 1:1 ratio. 5 mice from each group were culled at 2, 24, 48 and 72 hpi and each organ was individually collected, homogenised, and the ration of each marked strain determined. Species evenness was calculated per sample (Shannon’s diversity index (H) was calculated and then divided by the natural logarithm of species richness) and then linear mean regression was performed for both the wildtype and *pbp4* strain in each organ over time to compare correlations. The gradient of these lines was compared to determine a change in clonality compared to the wildtype strain.

### MDM survival

Monocytes were isolated from peripheral blood mononuclear cells (PBMCs) from healthy donors, as previously described [18]. Ficoll Plaque (GE Healthcare) density centrifugation was used to isolate PBMCs from donor blood, which were seeded into 24 well plates (Corning) at 2 x 10^6^ cells/ml in RPMI 1640 medium with the addition of 2 mM/l L-glutamine (Lonza) which was further supplemented with 10% v/v new-born foetal calf serum (Gibco). Wells contained approximately 2 x 10^5^ MDM. After incubating for 24 hours at 37°C with 5% CO2, cells were washed with fresh RPMI 1640 with 2 mM/l L-glutamine and with 10% v/v low endotoxin heat inactivated foetal calf serum (Biosera) to remove non-adherent cells. Cells had supernatant removed twice a week which was replaced with fresh supplemented RPMI 1640 and differentiated MDMs were used at 14 days post isolation. All media and reagents were warmed to 37°C prior to contact with MDMs (unless otherwise stated). MDMs were challenged with *S*. *aureus* strains at an MOI of 5 (i.e. 1 x 10^6^ CFU per well of 2 x 10^5^ MDMs) in RPMI-1640. MDMs were challenged with *S*. *aureus* for 4 hours at 37°C, 5% v/v CO_2_, after which infected media was removed and MDMs were washed with ice cold PBS. Extracellular bacteria were killed by the addition of 100 μg/ml gentamicin for 30 min in RPMI-1640 media. MDMs were then incubated in RPMI-1640 supplemented with 4 μg/ml gentamicin and 0.8 μg/ml lysostaphin until the desired time point. At the desired time points MDMs were washed with PBS and incubated for 12 min with 250 μl 2% w/v saponin at 37°C, 5% CO_2_. PBS was added to make well volume 1 ml and cells were further lysed by vigorous pipetting, with MDM lysis confirmed by light microscopy. Viable intracellular CFU was determined by serial dilution, and spotting 10 μl onto TSA plates, which were incubated at 37°C overnight. To confirm gentamicin killing of extracellular bacteria, control MDMs were fixed with 2% v/v paraformaldehyde before bacterial challenge, showing absence of bacteria in lysates after gentamicin treatment. Other control MDMs were not infected with *S*. *aureus* but otherwise treated as above, to confirm that all bacteria recovered came from the initial inoculum. All MDM experiments had two biological repeats for each time point and strain.

### Neutrophil survival

Neutrophil work followed a modified protocol previously described [18]. Neutrophils were purified from anti-coagulated human blood and kept at 37°C with 5% CO_2_ (v/v) in RPMI 1640 medium. Neutrophils were kept in 96 well Tissue Culture plates (Corning) containing 90 μl of approximately 2.5 x 10^6^ cells/ml (around 225,000 neutrophils per well). These were infected with 10 μl of bacterial stock culture to produce a MOI of 5. After 30 minutes, a sample to calculate phagocytosed bacteria numbers was collected and the remaining cells treated with gentamicin (to 40 μg/ml). After 60 and 120 min of co-culture, 100 μl of sample was transferred to a 1.5 ml Eppendorf tube and centrifuged for 3 min at 400 x g to recover neutrophils. Neutrophils were washed with 1 ml ice cold sterile PBS, and then lysed with 1 ml room temperature alkali water (6M NaOH added to dH_2_O until pH 11) and vigorous pipetting. Intracellular CFU were determined by serially diluting in sterile PBS and plating 10 μl spots onto TSA, which were left overnight at 37°C, and CFU calculated the next day by directly counting.

### Flow cytometry

Analysis of *S*. *aureus* particle size was analysed by measuring the forward scatter (FSC) of particles. *S*. *aureus* strains were cultured in 24 well plates at 37°C and 200 rpm until an OD_600_ of 0.6 (exponential phase) was achieved in 1 ml TSB. Bacteria were diluted 1:100 in PBS filtered through a 0.2 μm syringe filter. FSC was analysed by flow cytometry using a Millipore Guava EasyCyte system. Light scatter data were obtained with logarithmic amplifiers for 2500 events, and each strain was measured in triplicate, from three independent cultures.

### Statistical analysis

Statistical analysis was performed using Prism version 8.3.0 (GraphPad). Zebrafish experiments are representative of n = 2 unless otherwise stated, with figures showing combined results of replicate experiments. Comparison between survival curves was made using the log-rank (Mantel Cox) test. For bacterial count and weight change comparison in murine experiments the Mann-Whitney U test was used. For comparison of two or more independent samples simultaneously the Kruskal Wallis test was applied with Dunn’s multiple comparison test. Shannon’s diversity index was calculated using the equation $H=-\sum_{i=1}^{R}piln(pi),$ (where *H* = Shannon diversity index and *p*_*i*_ = the proportion of species *i* relative to the total number of species present). Species evenness was derived from the Shannon diversity index using the equation *EH* = *H*/ln*S* (where *E*_*H*_ = species evenness, H = Shannon diversity index and *S* = the total number of species within the community (species richness)). Species evenness within an organ over time was analysed using mean linear regression analysis. For relative fitness experiments, the equation $w=\frac{x_{2(1-x_{1})}}{x_{1(1-x_{2})}}$ was used and analysed using a one sample Wilcoxon signed rank test (w = relative fitness, X_1_ = starting mutant proportion and X_2_ = ending mutant proportion).

## Supporting information

S1 Fig Low magnification TEM images of *S*. *aureus* and murine kidney homogenate. Thin section of chemically fixed *S*. *aureus* NewHG *kan*^*R*^ (SJF 3680) cultured in TSB to **(A)** exponential or **(B)** stationary phase at low magnification (1900 x and 6800 x respectively). **(C)** NewHG *kan*^*R*^ (SJF 3680) recovered from murine kidneys 72 hpi (6800 x magnification). **(D)** low magnification (6800 x magnification) TEM images of processed uninfected murine kidney. Scale bar (blue line) represents 5000 nm for **(A)**, and 1000 nm for **(B), (C)** and **(D)**.
(PDF)

S2 Fig The infection dynamics *S*. *aureus pbp4* mutants. **(A)** Approximately 1500 CFU of bacteria (mutant or wildtype) was injected into the circulation valley of LWT zebrafish embryos around 30 hpf. Survival curve produced to compare the virulence of parental NewHG (SJF 3663, WT, black line) to NewHG *pbp4*::*ery* (SJF 5103) (3 repeats, n>20). **(B-D)** Mice (n = 10) were injected with approximately 1x10^7^ CFU *S*. *aureus* NewHG *kan*^*R*^ (WT, SJF 3680) or NewHG *pbp4*::*ery* (SJF 5103). **(B)** Weight loss 72 hpi and CFUs recovered from **(C)** livers (* p = 0.0294) and **(D)** kidneys were determined. Groups were compared using a Mann-Whitney U test (NewHG *kan*^*R*^–black circles, NewHG *pbp4*::*ery* blue squares). One mouse was found dead 72 hpi in the NewHG *kan*^*R*^ group and was excluded from the analysis. **(E-F)** Mice (n = 20) were injected with a 1:1 ratio (totalling approximately 1 x 10^7^ CFU) of two resistance marker tagged NewHG variants. 5 mice were culled at each time point and the CFU ratios in the liver, left kidney, right kidney, spleen, lungs and heart were determined. **(E)** The proportions of NewHG *kan*^*R*^ (SJF 3680, green) and NewHG *tet*^*R*^ (SJF 3681, blue) and **(F)** proportions of NewHG *kan*^*R*^ *pbp4*::*ery* (SJF 5136, green) and NewHG *tet*^*R*^ *pbp4*::*ery* (SJF 5135, blue) recovered at each time point from each organ in each mouse. The number in each pie chart represents the log number of bacteria recovered (i.e. 10^6^ CFU = 6). H.P.I: hours post infection, M.N.: Mouse number. Total CFU of recovered NewHG (black circles) and NewHG *pbp4*::*ery* (blue squares) strains from **(G)** liver, **(H) l**eft kidney, **(I)** right kidney, **(J)** spleen, **(K)** lungs and **(L)** heart. The population evenness from each mouse at different time points for NewHG *kan*^*R*^ (SJF 3680) and NewHG *tet*^*R*^ (SJF 3681) (black circles and lines) and NewHG *kan*^*R*^ *pbp4*::*ery* (SJF 5136) and NewHG *tet*^*R*^ *pbp4*::*ery* (SJF 5135) (blue squares and lines) for **(M)** livers, **(N)** left kidney, **(O)** right kidney, **(P)** spleen, **(Q)** lungs and **(R)** heart. Lines are mean linear regression, which were calculated and compared using Prism software. All linear regressions were found to be non-significant, so the slopes of the lines are not significantly different from one another. Livers p = 0.6077, Spleen p = 0.2339, Left kidney: p = 0.7691, Right kidney p = 0.6604, Lungs p = 0.6758 and Heart p = 0.9300. **(S)** The number of internalised NewHG *kan*^*R*^ (SJF 3680, black bars) and NewHG *pbp4*::*ery* (SJF 5103, blue bars) and the number remaining in the extracellular supernatant after 30 min of co-incubation with human neutrophils. Error bars represent the standard deviation of the mean. (n = 4, each consisting of 3 intra-assay repeats). Results analysed with a one-way ANOVA with Tukey’s multiple comparison post-test. **(T)** Intracellular NewHG *kan*^*R*^ (SJF 3680, black circles) and NewHG *pbp4*::*ery* (SJF 5103, blue squares) CFU after co-incubation with neutrophils for 60 or 120 minutes. (n = 4, each consisting of 3 intra-assay repeats). Error bars represent the mean and standard deviation of the mean. Results analysed with a two-way ANOVA with Tukey’s correction.
(PDF)

S3 Fig The role of *sagB* in the zebrafish embryo model. **(A)** Survival curves showing the attenuation of SH1000 *sagB*::*kan* (SJF 4608, red line) compared to SH1000 (SJF 682, black lines) (3 repeats, n>20, **** p < 0.0001) and **(B)** the attenuation of NewHG *sagB*::*kan* (SJF 4912, red line) compared to parental NewHG (SJF 3663, black lines) (2 repeats, n>20, ** p = 0.0043). **(C, D)** Bacterial CFU were recovered from zebrafish embryos infected with 1500 CFU **(C)** SH1000 (SJF 682) or **(D)** SH1000 *sagB*::*kan* (SJF 4608) (n = 50–60) or **(E)** NewHG (SJF 3663) or **(F)** NewHG *sagB*::*kan* (SJF 4912) (n = 70–85) at times shown. Open black circles are live embryos and red circles are dead embryos.
(PDF)

S4 Fig Augmentation of *S*. *aureus* strains using staphylococcal peptidoglycan in the murine sepsis model. Approximately 1x10^6^ CFU of either *S*. *aureus* NewHG *kan*^*R*^ (WT, SJF 3680), NewHG *pbp4*::*ery* (SJF 5103) or NewHG *sagB*::*kan* (SJF 4912) with or without 250 μg WT *S*. *aureus* PG injected intravenously into mice (n = 5). **(A, E)** Weight loss 72 hpi (* p = 0.0159, ** p = 0.0079) and CFUs recovered from **(B, F)** livers (* p = 0.0397, ** p = 0.0079) **(C, G)** kidneys and **(D, H)** spleen were determined. Groups were compared using Mann-Whitney U tests (NewHG *kan*^*R*^–black circles, NewHG *pbp4*::*ery—*blue squares, NewHG *sagB*::*kan–*red squares). One mouse (infected with NewHG *pbp4*::*ery* and 250 μg PG) was culled at 56 hpi due to reaching severity limits, so was culled. This data is represented as a green diamond but has been excluded from statistical analysis.
(PDF)

S5 Fig Growth and virulence of NewHG *sagB pbp4 in vitro* and in the murine sepsis model. **(A)** Growth of parental NewHG (SJF 3663, black circles) in TSB compared to: NewHG *sagB*::*kan* (SJF 4912, red squares), NewHG *pbp4*::*ery* (SJF 5103, blue diamonds) and NewHG *sagB*::*kan pbp4*::*ery* (SJF 5147, purple triangles). Bacterial cultures were prepared in triplicate and error bars represent the standard deviation of the mean. Mice (n = 10) were injected intravenously with approximately 1x10^7^ CFU *S*. *aureus* NewHG *kan*^*R*^ (WT, SJF 3680), NewHG *sagB*::*kan* (SJF 4912), NewHG *pbp4*::*ery* (SJF 5103) or NewHG *sagB*::*kan pbp4*::*ery* (SJF 5147). CFUs recovered from **(B)** spleens, **(C)** lungs and **(D)** hearts were determined. Groups were compared using a Mann-Whitney U test (NewHG *kan*^*R*^–black circles, NewHG *sagB*::*kan–*red squares, NewHG *pbp4*::*ery* blue diamonds, NewHG *sagB*::*kan pbp4*::*ery–*purple triangles).
(PDF)

S6 Fig Growth and virulence of *S*. *aureus* glucosaminidase mutants. Growth (measured by OD_600_ and CFU) of parental SH1000 (SJF 682, black circles solid line) or sonicated SH1000 (open black circles and broken line) in TSB compared to: **(A, B)** SH1000 *atl* (SJF 1367, yellow squares) and sonicated SH1000 *atl* (yellow open squares, broken lines), **(D, E)** SH1000 *sagA* (SJF 4606, blue squares) and sonicated SH1000 *sagA* (blue open squares, broken lines), **(G, H)** SH1000 *scaH* (SJF 4607, red squares) and sonicated SH1000 *scaH* (red open squares, broken lines), **(J, K)** SH1000 *atl sagA* (SJF 5261, green squares) and sonicated SH1000 *atl sagA* (green open squares, broken lines), **(L, M)** SH1000 *atl scaH* (SJF 5262, orange squares) and sonicated SH1000 *atl scaH* (orange open squares, broken lines), **(N, O)** SH1000 *sagA scaH* (SJF 5217, purple squares) and sonicated SH1000 *sagA scaH* (purple open squares, broken lines). Bacterial cultures were prepared in triplicate and error bars represent the standard deviation of the mean. Sonicated strains were sonicated for 20 seconds at an amplitude of 5 microns. Survival curves of zebrafish embryos injected with approximately 1500 CFU of *S*. *aureus* SH1000 (SJF 682, black lines) or **(C)** approximately 500 CFU SH1000 *atl* (1500 CFU after sonication) (SJF 1367, yellow line) **(F)** approximately 1500 CFU SH1000 *sagA* (SJF 4606, blue line) or **(I)** approximately 1500 CFU SH1000 *scaH* (SJF 4607, red line). (3 repeats, n>20), all groups are not significantly different from the parental SH1000 strain.
(PDF)

S7 Fig The role of Atl, SagA and ScaH in *S*. *aureus* growth and virulence. **(A,B)** Growth of parental SH1000 (SJF 682, black circles solid line) or sonicated SH1000 (open black circles and broken line) in TSB compared to SH1000 *atl sagA scaH* (SJF 4611, brown squares) and sonicated SH1000 *atl sagA scaH* (brown open squares, broken lines). Organ homogenates from Fig 7G and 7H were sonicated to get a better representation of bacterial load in the **(C)** livers (p values on graph) and **(D)** kidneys (* p = 0.0364, *** p = 0.0009). Groups were compared using a Kruskal-Wallis test with multiple comparisons (unsonicated SH1000 *kan*^*R*^–black circles, sonicated SH1000 *kan*^*R*^–open black circles, unsonicated SH1000 *atl sagA scaH—*brown squares and sonicated SH1000 *atl sagA scaH–*open brown squares). Strains and organ homogenates were sonicated for 20 seconds at an amplitude of 5 microns. **(E-J)** Mice (n = 5) were injected with approximately 1x10^7^ CFU sonicated *S*. *aureus* SH1000 *atl sagA scaH* (SJF 4611) alone, or with 250 μg SH1000 PG (SJF 682, WT PG) or 250 μg SH1000 *atl sagA scaH* PG (mutant PG). **(E)** Weight loss 72 hpi (** p = 0.0079, * p = 0.0159) and CFUs recovered from **(F)** livers (* p = 0.0159), **(G)** kidneys (p values on graph), **(H)** spleen, **(I)** lungs and **(J)** heart were determined. Groups were compared using a Mann-Whitney U test. (SH1000 *atl sagA scaH* only–black circles, SH1000 *atl sagA scaH* with wildtype PG—red squares SH1000 *atl sagA scaH* with mutant PG–blue triangles). One mouse in the SH1000 *atl sagA scaH* and WT PG group reached severity limits at 48 hpi and was culled and has been excluded from analysis.
(PDF)

S1 Table *S*. *aureus* strains used in this study. (PDF)

S2 Table Oligonucleotides used in this study. (PDF)

S3 Table Muropeptide database. Identity of *S*. *aureus* muropeptides with numbering system used as a part of this study. The observed retention times, mass and charge states are also noted.
(PDF)

S4 Table Raw integration results. Identified and measured peaks of **(A-C)** exponential phase *S*. *aureus*, **(D-F)** stationary phase *S*. *aureus* and **(G-H)** murine infection derived *S*. *aureus*.
(PDF)
