# Supplementary material for: Staphylococcus aureus cell wall structure and dynamics during host-pathogen interaction
Source: PLoS Pathog. 2021 Mar 31;17(3):e1009468. doi: 10.1371/journal.ppat.1009468 (PMC8041196; doi:10.1371/journal.ppat.1009468)
Supplement: S1 Fig — Thin section of chemically fixed S. aureus NewHG kanR (SJF 3680) cultured in TSB to (A) exponential or (B) stationary phase at low magnification (1900 x and 6800 x respectively). (C) NewHG kanR (SJF 3680) recovered from murine kidneys 72 hpi (6800 x magnification). (D) low magnification (6800 x magnification) TEM images of processed uninfected murine kidney. Scale bar (blue line) represents 5000 nm for (A), and 1000 nm for (B), (C) and (D). (PDF) [file ppat.1009468.s001.pdf]

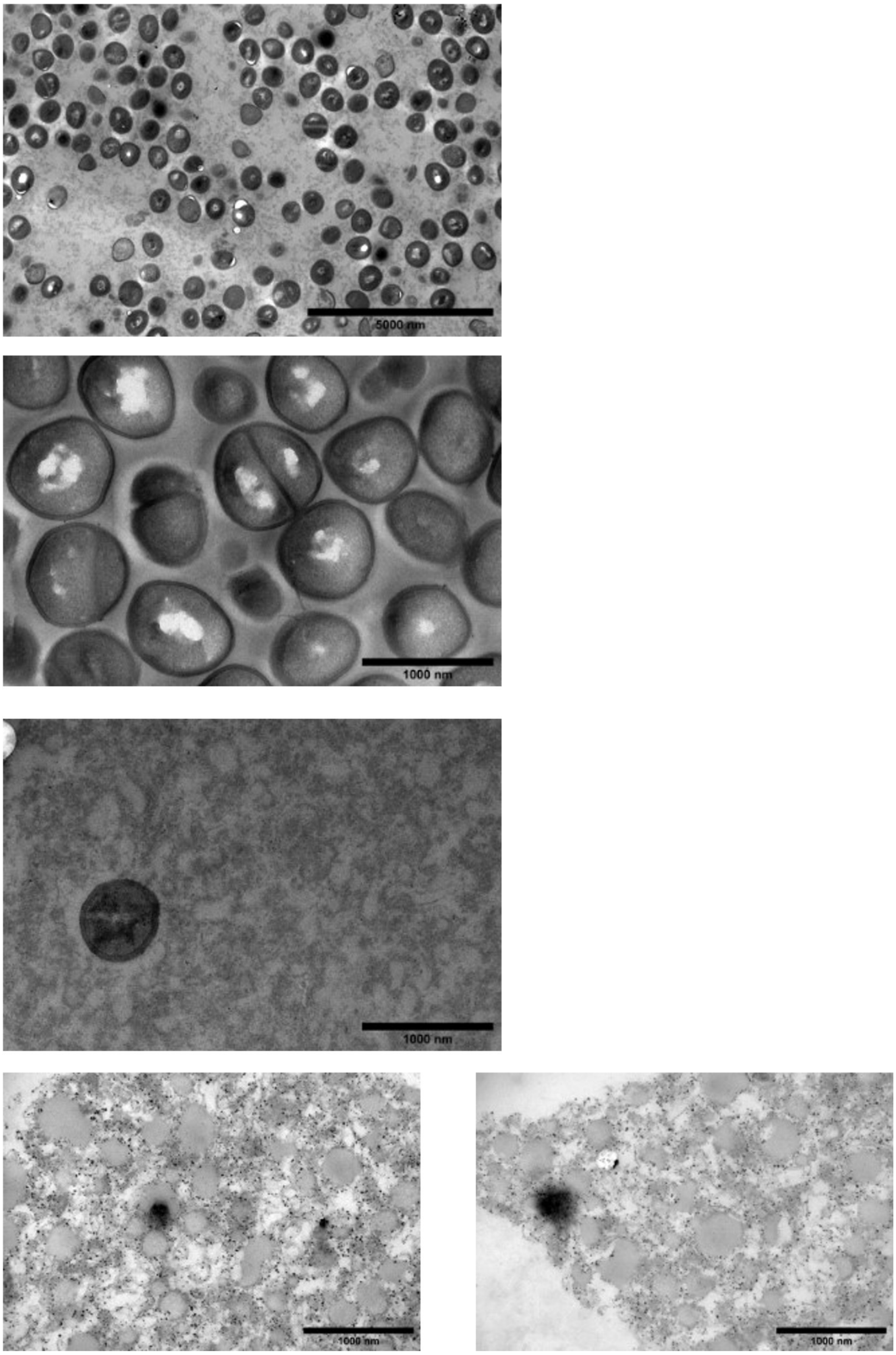

S1 Fig. Low magnification TEM images of *S. aureus* and murine kidney homogenate.

Thin section of chemically fixed *S. aureus* NewHG *kan*^{R} (SJF 3680) cultured in TSB to **(A)** exponential or **(B)** stationary phase at low magnification (1900 x and 6800 x respectively). **(C)** NewHG *kan*^{R} (SJF 3680) recovered from murine kidneys 72 hpi (6800 x magnification). **(D)** low magnification (6800 x

magnification) TEM images of processed uninfected murine kidney. Scale bar (blue line) represents 5000 nm for **(A)**, and 1000 nm for **(B), (C)** and **(D)**.
