# Supplementary material for: Staphylococcus aureus cell wall structure and dynamics during host-pathogen interaction
Source: PLoS Pathog. 2021 Mar 31;17(3):e1009468. doi: 10.1371/journal.ppat.1009468 (PMC8041196; doi:10.1371/journal.ppat.1009468)
Supplement: S2 Fig — (A) Approximately 1500 CFU of bacteria (mutant or wildtype) was injected into the circulation valley of LWT zebrafish embryos around 30 hpf. Survival curve produced to compare the virulence of parental NewHG (SJF 3663, WT, black line) to NewHG pbp4::ery (SJF 5103) (3 repeats, n>20). (B-D) Mice (n = 10) were injected with approximately 1x10^7^ CFU S. aureus NewHG kanR (WT, SJF 3680) or NewHG pbp4::ery (SJF 5103). (B) Weight loss 72 hpi and CFUs recovered from (C) livers (* p = 0.0294) and (D) kidneys were determined. Groups were compared using a Mann-Whitney U test (NewHG kanR–black circles, NewHG pbp4::ery blue squares). One mouse was found dead 72 hpi in the NewHG kanR group and was excluded from the analysis. (E-F) Mice (n = 20) were injected with a 1:1 ratio (totalling approximately 1 x 10^7^ CFU) of two resistance marker tagged NewHG variants. 5 mice were culled at each time point and the CFU ratios in the liver, left kidney, right kidney, spleen, lungs and heart were determined. (E) The proportions of NewHG kanR (SJF 3680, green) and NewHG tetR (SJF 3681, blue) and (F) proportions of NewHG kanR pbp4::ery (SJF 5136, green) and NewHG tetR pbp4::ery (SJF 5135, blue) recovered at each time point from each organ in each mouse. The number in each pie chart represents the log number of bacteria recovered (i.e. 106 CFU = 6). H.P.I: hours post infection, M.N.: Mouse number. Total CFU of recovered NewHG (black circles) and NewHG pbp4::ery (blue squares) strains from (G) liver, (H) left kidney, (I) right kidney, (J) spleen, (K) lungs and (L) heart. The population evenness from each mouse at different time points for NewHG kanR (SJF 3680) and NewHG tetR (SJF 3681) (black circles and lines) and NewHG kanR pbp4::ery (SJF 5136) and NewHG tetR pbp4::ery (SJF 5135) (blue squares and lines) for (M) livers, (N) left kidney, (O) right kidney, (P) spleen, (Q) lungs and (R) heart. Lines are mean linear regression, which were calculated and compared using Prism software. All linear regre [file ppat.1009468.s002.pdf]

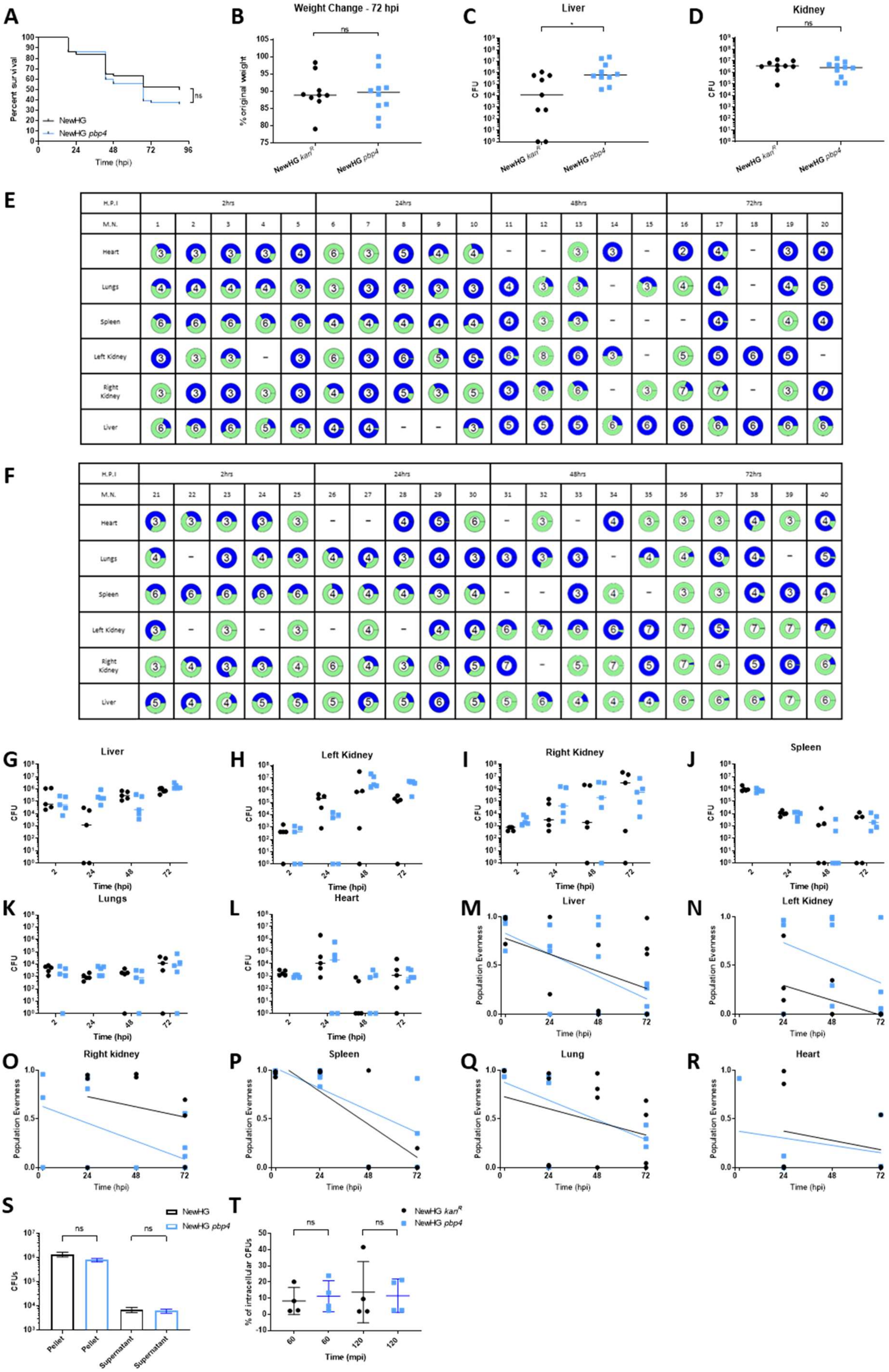

S2 Fig. The infection dynamics *S. aureus pbp4* mutants.

(A) Approximately 1500 CFU of bacteria (mutant or wildtype) was injected into the circulation valley of LWT zebrafish embryos around 30 hpf. Survival curve produced to compare the virulence of parental NewHG (SJF 3663, WT, black line) to NewHG *pbp4::ery* (SJF 5103) (3 repeats, n>20). **(B-D)** Mice (n = 10) were injected with approximately 1x10^7 CFU *S. aureus* NewHG *kan*^{R} (WT, SJF 3680) or NewHG *pbp4::ery* (SJF 5103). **(B)** Weight loss 72 hpi and CFUs recovered from **(C)** livers (* p = 0.0294) and **(D)** kidneys were determined. Groups were compared using a Mann-Whitney U test (NewHG *kan*^{R} – black circles, NewHG *pbp4::ery* blue squares). One mouse was found dead 72 hpi in the NewHG *kan*^{R} group and was excluded from the analysis. **(E-F)** Mice (n = 20) were injected with a 1:1 ratio (totalling approximately 1 x 10^7 CFU) of two resistance marker tagged NewHG variants. 5 mice were culled at each time point and the CFU ratios in the liver, left kidney, right kidney, spleen, lungs and heart were determined. **(E)** The proportions of NewHG *kan*^{R} (SJF 3680, green) and NewHG *tet*^{R} (SJF 3681, blue) and **(F)** proportions of NewHG *kan*^{R} *pbp4::ery* (SJF 5136, green) and NewHG *tet*^{R} *pbp4::ery* (SJF 5135, blue) recovered at each time point from each organ in each mouse. The number in each pie chart represents the log number of bacteria recovered (i.e. 10^6 CFU = 6). H.P.I: hours post infection, M.N.: Mouse number. Total CFU of recovered NewHG (black circles) and NewHG *pbp4::ery* (blue squares) strains from **(G)** liver, **(H) l**eft kidney, **(I)** right kidney, **(J)** spleen, **(K)** lungs and **(L)** heart. The population evenness from each mouse at different time points for NewHG *kan*^{R} (SJF 3680) and NewHG *tet*^{R} (SJF 3681) (black circles and lines) and NewHG *kan*^{R} *pbp4::ery* (SJF 5136) and NewHG *tet*^{R} *pbp4::ery* (SJF 5135) (blue squares and lines) for **(M)** livers, **(N)** left kidney, **(O)** right kidney, **(P)** spleen, **(Q)** lungs and **(R)** heart. Lines are mean linear regression, which were calculated and compared using Prism software. All linear regressions were found to be non-significant, so the slopes of the lines are not significantly different from one another. Livers p = 0.6077, Spleen p = 0.2339, Left kidney: p = 0.7691, Right kidney p = 0.6604, Lungs p = 0.6758 and Heart p = 0.9300. **(S)** The number of internalised NewHG *kan*^{R} (SJF 3680, black bars) and NewHG *pbp4::ery* (SJF 5103, blue bars) and the number remaining in the extracellular supernatant after 30 min of co-incubation with human neutrophils. Error bars represent the standard deviation of the mean. (n = 4, each consisting of 3 intra-assay repeats). Results analysed with a one-way ANOVA with Tukey's multiple comparison post-test. **(T)** Intracellular NewHG *kan*^{R} (SJF 3680, black circles) and NewHG *pbp4::ery* (SJF 5103, blue squares) CFU after co-incubation with neutrophils for 60 or 120 minutes. (n = 4, each consisting of 3 intra-assay repeats). Error bars represent the mean and standard deviation of the mean. Results analysed with a two-way ANOVA with Tukey's correction.
