# Supplementary material for: Staphylococcus aureus cell wall structure and dynamics during host-pathogen interaction
Source: PLoS Pathog. 2021 Mar 31;17(3):e1009468. doi: 10.1371/journal.ppat.1009468 (PMC8041196; doi:10.1371/journal.ppat.1009468)
Supplement: S3 Fig — (A) Survival curves showing the attenuation of SH1000 sagB::kan (SJF 4608, red line) compared to SH1000 (SJF 682, black lines) (3 repeats, n>20, **** p < 0.0001) and (B) the attenuation of NewHG sagB::kan (SJF 4912, red line) compared to parental NewHG (SJF 3663, black lines) (2 repeats, n>20, ** p = 0.0043). (C, D) Bacterial CFU were recovered from zebrafish embryos infected with 1500 CFU (C) SH1000 (SJF 682) or (D) SH1000 sagB::kan (SJF 4608) (n = 50–60) or (E) NewHG (SJF 3663) or (F) NewHG sagB::kan (SJF 4912) (n = 70–85) at times shown. Open black circles are live embryos and red circles are dead embryos. (PDF) [file ppat.1009468.s003.pdf]

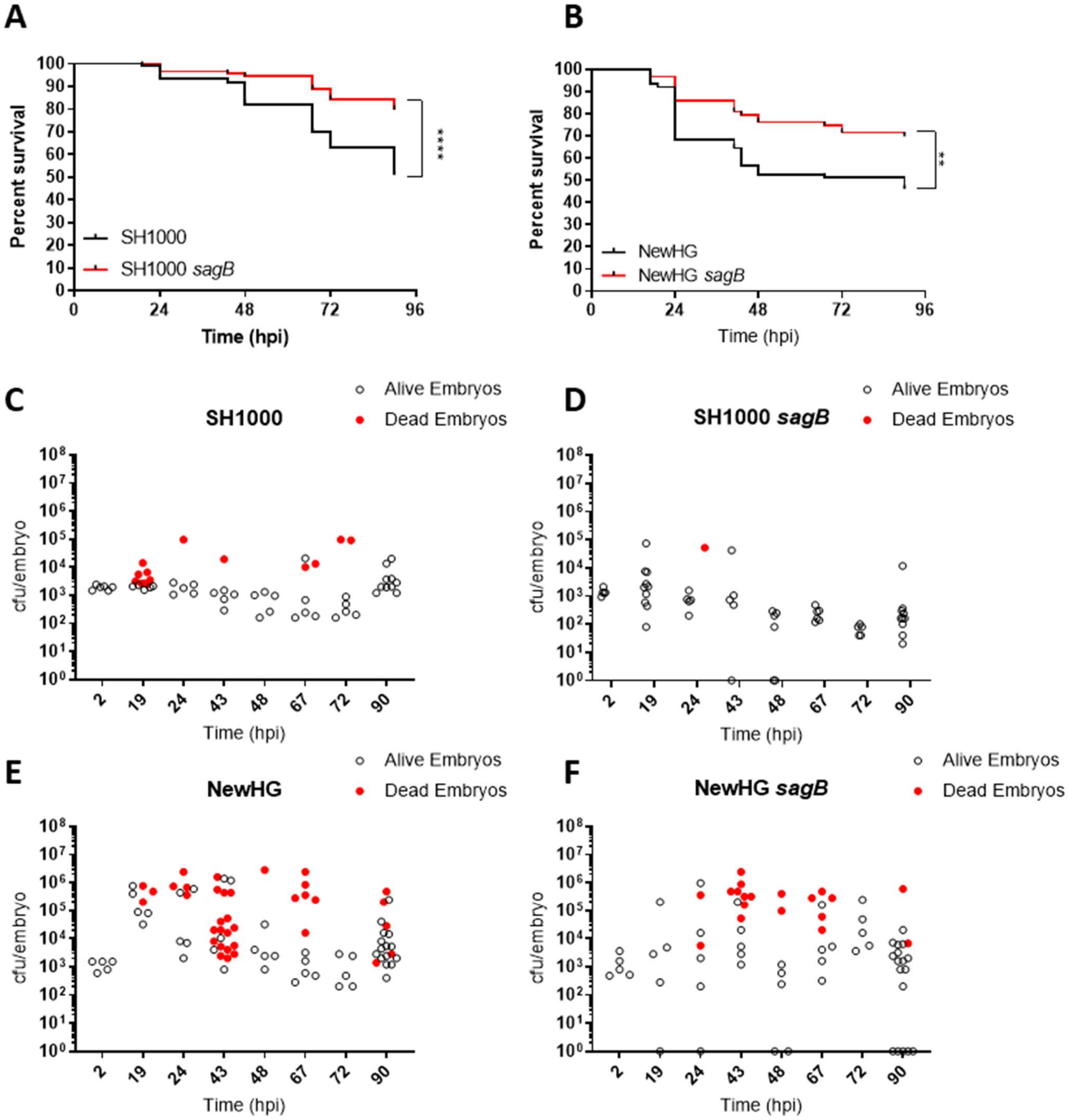

S3 Fig. The role of *sagB* in the zebrafish embryo model.

(A) Survival curves showing the attenuation of SH1000 *sagB::kan* (SJF 4608, red line) compared to SH1000 (SJF 682, black lines) (3 repeats, n>20, **** $p < 0.0001$) and **(B)** the attenuation of NewHG *sagB::kan* (SJF 4912, red line) compared to parental NewHG (SJF 3663, black lines) (2 repeats, n>20, ** $p = 0.0043$). **(C, D)** Bacterial CFU were recovered from zebrafish embryos infected with 1500 CFU **(C)** SH1000 (SJF 682) or **(D)** SH1000 *sagB::kan* (SJF 4608) (n= 50-60) or **(E)** NewHG (SJF 3663) or **(F)** NewHG *sagB::kan* (SJF 4912) (n= 70-85) at times shown. Open black circles are live embryos and red circles are dead embryos.
