# Supplementary material for: Staphylococcus aureus cell wall structure and dynamics during host-pathogen interaction
Source: PLoS Pathog. 2021 Mar 31;17(3):e1009468. doi: 10.1371/journal.ppat.1009468 (PMC8041196; doi:10.1371/journal.ppat.1009468)
Supplement: S4 Fig — Approximately 1x10^6^ CFU of either S. aureus NewHG kanR (WT, SJF 3680), NewHG pbp4::ery (SJF 5103) or NewHG sagB::kan (SJF 4912) with or without 250 μg WT S. aureus PG injected intravenously into mice (n = 5). (A, E) Weight loss 72 hpi (* p = 0.0159, ** p = 0.0079) and CFUs recovered from (B, F) livers (* p = 0.0397, ** p = 0.0079) (C, G) kidneys and (D, H) spleen were determined. Groups were compared using Mann-Whitney U tests (NewHG kanR–black circles, NewHG pbp4::ery—blue squares, NewHG sagB::kan–red squares). One mouse (infected with NewHG pbp4::ery and 250 μg PG) was culled at 56 hpi due to reaching severity limits, so was culled. This data is represented as a green diamond but has been excluded from statistical analysis. (PDF) [file ppat.1009468.s004.pdf]

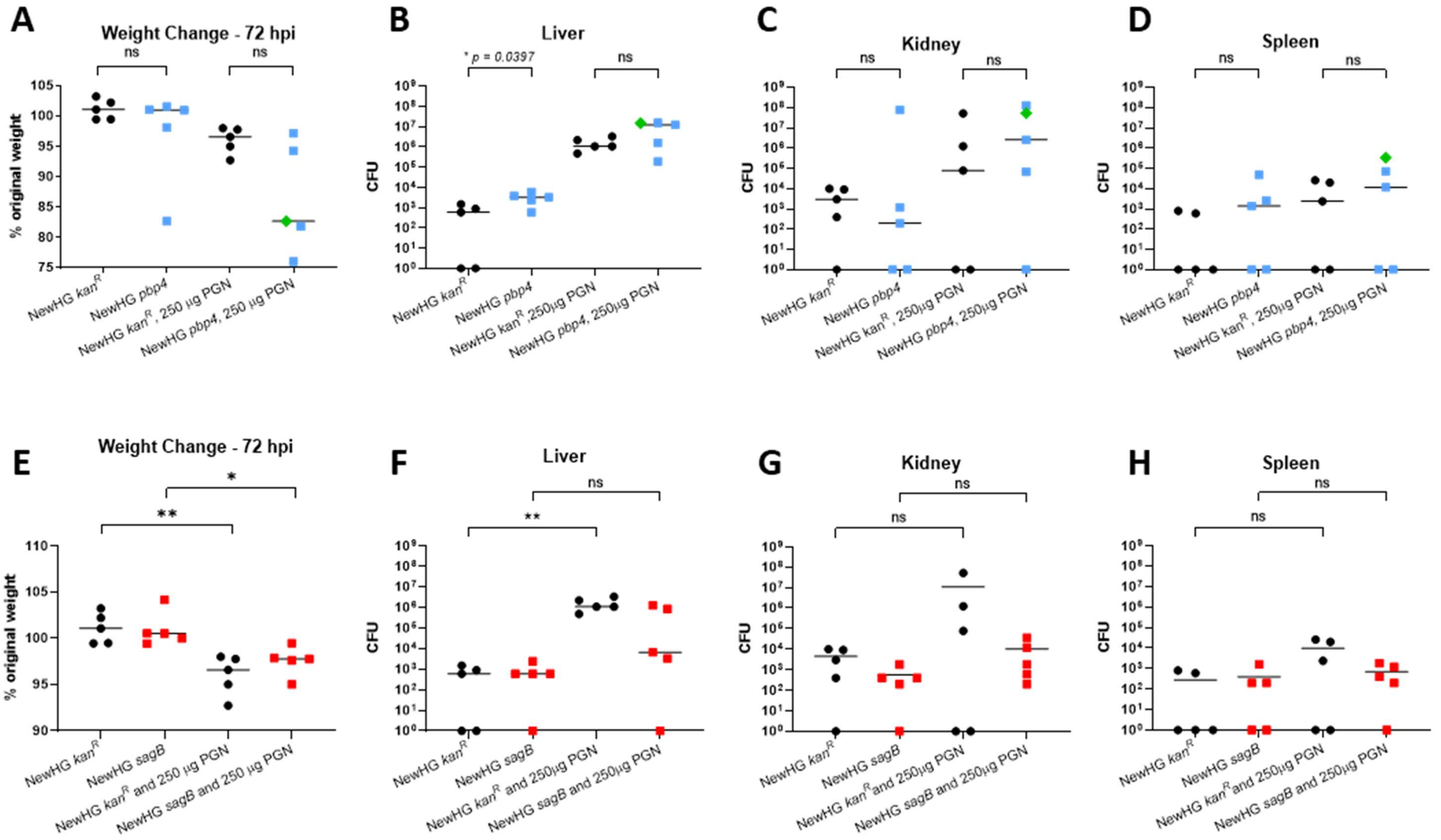

S4 Fig. Augmentation of *S. aureus* strains using staphylococcal peptidoglycan in the murine sepsis model.

Approximately 1x10^6 CFU of either *S. aureus* NewHG *kan^R* (WT, SJF 3680)*,* NewHG *pbp4::ery* (SJF 5103) or NewHG *sagB::kan* (SJF 4912) with or without 250 µg WT *S. aureus* PG injected intravenously into mice (n=5). **(A, E)** Weight loss 72 hpi (* p = 0.0159, ** p = 0.0079) and CFUs recovered from **(B, F)** livers (* p = 0.0397, ** p = 0.0079) **(C, G)** kidneys and **(D, H)** spleen were determined. Groups were compared using Mann-Whitney U tests (NewHG *kan^R* – black circles, NewHG *pbp4::ery* - blue squares, NewHG *sagB::kan* – red squares). One mouse (infected with NewHG *pbp4::ery* and 250 µg PG) was culled at 56 hpi due to reaching severity limits, so was culled. This data is represented as a green diamond but has been excluded from statistical analysis.
