# Supplementary material for: Staphylococcus aureus cell wall structure and dynamics during host-pathogen interaction
Source: PLoS Pathog. 2021 Mar 31;17(3):e1009468. doi: 10.1371/journal.ppat.1009468 (PMC8041196; doi:10.1371/journal.ppat.1009468)
Supplement: S5 Fig — (A) Growth of parental NewHG (SJF 3663, black circles) in TSB compared to: NewHG sagB::kan (SJF 4912, red squares), NewHG pbp4::ery (SJF 5103, blue diamonds) and NewHG sagB::kan pbp4::ery (SJF 5147, purple triangles). Bacterial cultures were prepared in triplicate and error bars represent the standard deviation of the mean. Mice (n = 10) were injected intravenously with approximately 1x10^7^ CFU S. aureus NewHG kanR (WT, SJF 3680), NewHG sagB::kan (SJF 4912), NewHG pbp4::ery (SJF 5103) or NewHG sagB::kan pbp4::ery (SJF 5147). CFUs recovered from (B) spleens, (C) lungs and (D) hearts were determined. Groups were compared using a Mann-Whitney U test (NewHG kanR–black circles, NewHG sagB::kan–red squares, NewHG pbp4::ery blue diamonds, NewHG sagB::kan pbp4::ery–purple triangles). (PDF) [file ppat.1009468.s005.pdf]

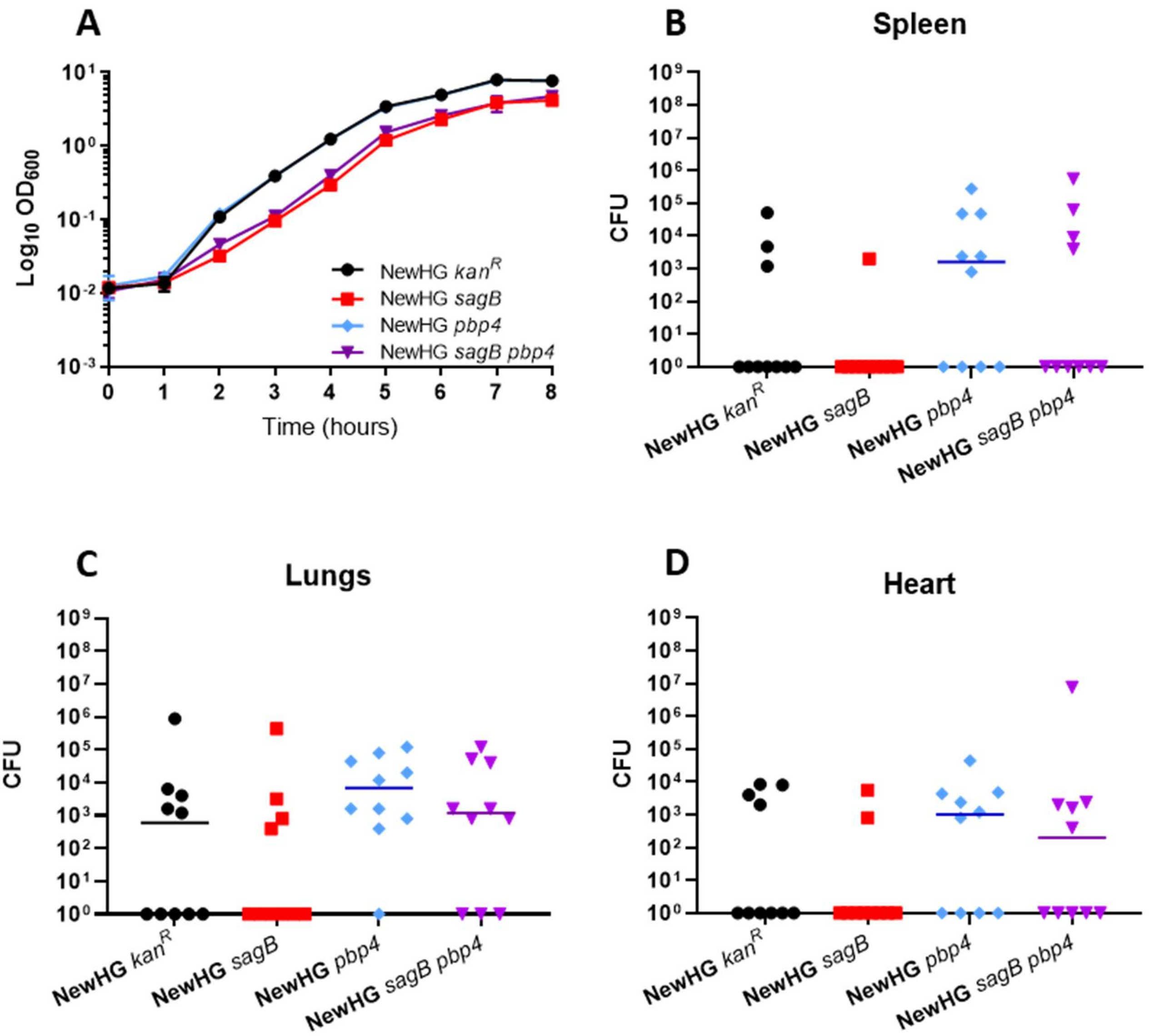

S5 Fig. Growth and virulence of NewHG *sagB pbp4 in vitro* and in the murine sepsis model.

(A) Growth of parental NewHG (SJF 3663, black circles) in TSB compared to: NewHG *sagB::kan* (SJF 4912, red squares), NewHG *pbp4::ery* (SJF 5103, blue diamonds) and NewHG *sagB::kan pbp4::ery* (SJF 5147, purple triangles). Bacterial cultures were prepared in triplicate and error bars represent the standard deviation of the mean. Mice (n = 10) were injected intravenously with approximately 1×10^7 CFU *S. aureus* NewHG *kan*^{R} (WT, SJF 3680), NewHG *sagB::kan* (SJF 4912), NewHG *pbp4::ery* (SJF 5103) or NewHG *sagB::kan pbp4::ery* (SJF 5147). CFUs recovered from **(B)** spleens, **(C)** lungs and **(D)** hearts were determined. Groups were compared using a Mann-Whitney U test (NewHG *kan*^{R} – black circles, NewHG *sagB::kan* – red squares, NewHG *pbp4::ery* blue diamonds, NewHG *sagB::kan pbp4::ery* – purple triangles).
