# Supplementary material for: Staphylococcus aureus cell wall structure and dynamics during host-pathogen interaction
Source: PLoS Pathog. 2021 Mar 31;17(3):e1009468. doi: 10.1371/journal.ppat.1009468 (PMC8041196; doi:10.1371/journal.ppat.1009468)
Supplement: S6 Fig — Growth (measured by OD600 and CFU) of parental SH1000 (SJF 682, black circles solid line) or sonicated SH1000 (open black circles and broken line) in TSB compared to: (A, B) SH1000 atl (SJF 1367, yellow squares) and sonicated SH1000 atl (yellow open squares, broken lines), (D, E) SH1000 sagA (SJF 4606, blue squares) and sonicated SH1000 sagA (blue open squares, broken lines), (G, H) SH1000 scaH (SJF 4607, red squares) and sonicated SH1000 scaH (red open squares, broken lines), (J, K) SH1000 atl sagA (SJF 5261, green squares) and sonicated SH1000 atl sagA (green open squares, broken lines), (L, M) SH1000 atl scaH (SJF 5262, orange squares) and sonicated SH1000 atl scaH (orange open squares, broken lines), (N, O) SH1000 sagA scaH (SJF 5217, purple squares) and sonicated SH1000 sagA scaH (purple open squares, broken lines). Bacterial cultures were prepared in triplicate and error bars represent the standard deviation of the mean. Sonicated strains were sonicated for 20 seconds at an amplitude of 5 microns. Survival curves of zebrafish embryos injected with approximately 1500 CFU of S. aureus SH1000 (SJF 682, black lines) or (C) approximately 500 CFU SH1000 atl (1500 CFU after sonication) (SJF 1367, yellow line) (F) approximately 1500 CFU SH1000 sagA (SJF 4606, blue line) or (I) approximately 1500 CFU SH1000 scaH (SJF 4607, red line). (3 repeats, n>20), all groups are not significantly different from the parental SH1000 strain. (PDF) [file ppat.1009468.s006.pdf]

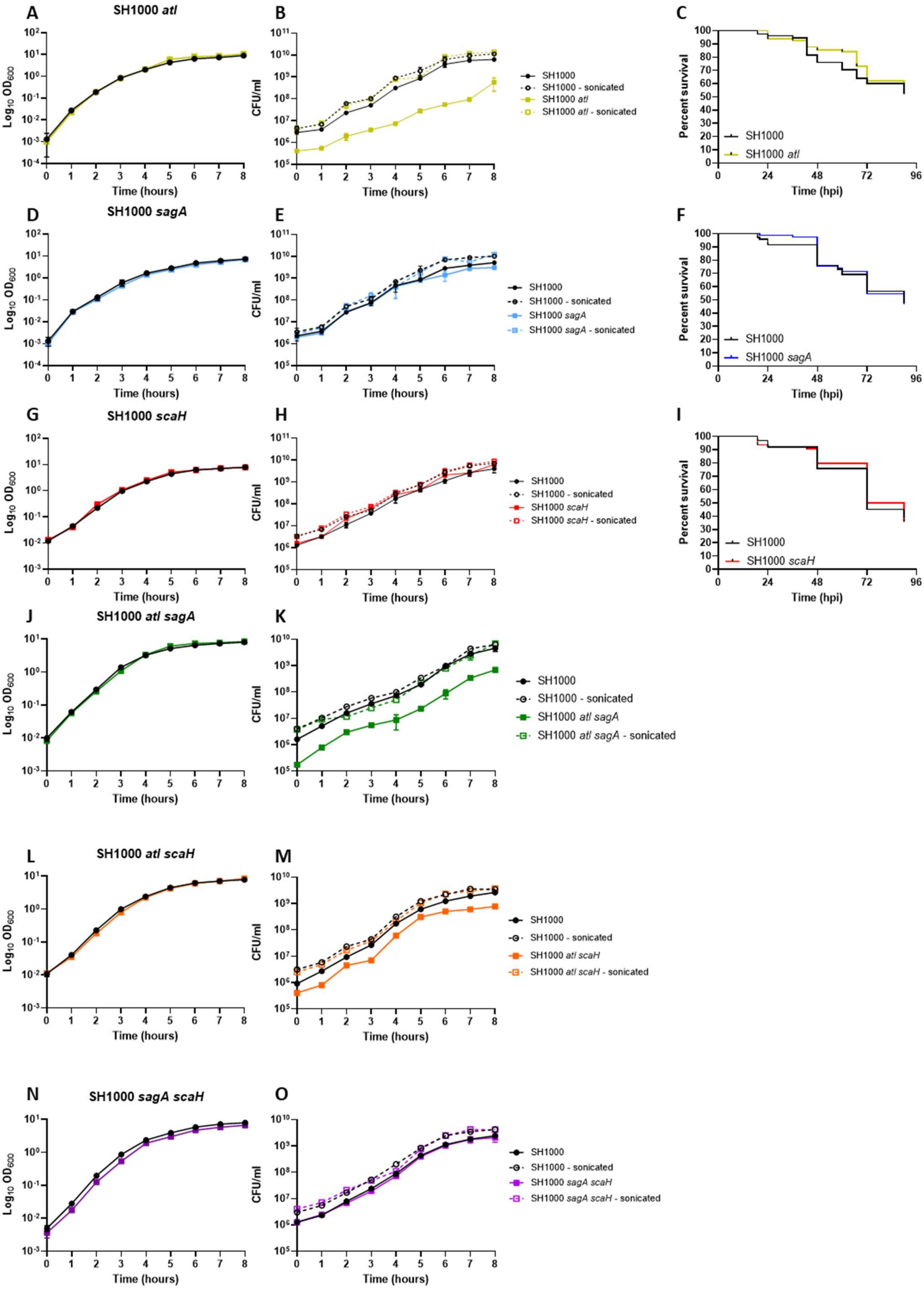

S6 Fig. Growth and virulence of *S. aureus* glucosaminidase mutants.

Growth (measured by OD_{600} and CFU) of parental SH1000 (SJF 682, black circles solid line) or sonicated SH1000 (open black circles and broken line) in TSB compared to: **(A, B)** SH1000 *atl* (SJF 1367, yellow squares) and sonicated SH1000 *atl* (yellow open squares, broken lines), **(D, E)** SH1000 *sagA* (SJF 4606, blue squares) and sonicated SH1000 *sagA* (blue open squares, broken lines), **(G, H)** SH1000 *scaH* (SJF 4607, red squares) and sonicated SH1000 *scaH* (red open squares, broken lines), **(J, K)** SH1000 *atl sagA* (SJF 5261, green squares) and sonicated SH1000 *atl sagA* (green open squares, broken lines), **(L, M)** SH1000 *atl scaH* (SJF 5262, orange squares) and sonicated SH1000 *atl scaH* (orange open squares, broken lines), **(N, O)** SH1000 *sagA scaH* (SJF 5217, purple squares) and sonicated SH1000 *sagA scaH* (purple open squares, broken lines). Bacterial cultures were prepared in triplicate and error bars represent the standard deviation of the mean. Sonicated strains were sonicated for 20 seconds at an amplitude of 5 microns. Survival curves of zebrafish embryos injected with approximately 1500 CFU of *S. aureus* SH1000 (SJF 682, black lines) or **(C)** approximately 500 CFU SH1000 *atl* (1500 CFU after sonication) (SJF 1367, yellow line) **(F)** approximately 1500 CFU SH1000 *sagA* (SJF 4606, blue line) or **(I)** approximately 1500 CFU SH1000 *scaH* (SJF 4607, red line). (3 repeats, n>20), all groups are not significantly different from the parental SH1000 strain.
