# Supplementary material for: Staphylococcus aureus cell wall structure and dynamics during host-pathogen interaction
Source: PLoS Pathog. 2021 Mar 31;17(3):e1009468. doi: 10.1371/journal.ppat.1009468 (PMC8041196; doi:10.1371/journal.ppat.1009468)
Supplement: S7 Fig — (A,B) Growth of parental SH1000 (SJF 682, black circles solid line) or sonicated SH1000 (open black circles and broken line) in TSB compared to SH1000 atl sagA scaH (SJF 4611, brown squares) and sonicated SH1000 atl sagA scaH (brown open squares, broken lines). Organ homogenates from Fig 7G and 7H were sonicated to get a better representation of bacterial load in the (C) livers (p values on graph) and (D) kidneys (* p = 0.0364, *** p = 0.0009). Groups were compared using a Kruskal-Wallis test with multiple comparisons (unsonicated SH1000 kanR–black circles, sonicated SH1000 kanR–open black circles, unsonicated SH1000 atl sagA scaH—brown squares and sonicated SH1000 atl sagA scaH–open brown squares). Strains and organ homogenates were sonicated for 20 seconds at an amplitude of 5 microns. (E-J) Mice (n = 5) were injected with approximately 1x10^7^ CFU sonicated S. aureus SH1000 atl sagA scaH (SJF 4611) alone, or with 250 μg SH1000 PG (SJF 682, WT PG) or 250 μg SH1000 atl sagA scaH PG (mutant PG). (E) Weight loss 72 hpi (** p = 0.0079, * p = 0.0159) and CFUs recovered from (F) livers (* p = 0.0159), (G) kidneys (p values on graph), (H) spleen, (I) lungs and (J) heart were determined. Groups were compared using a Mann-Whitney U test. (SH1000 atl sagA scaH only–black circles, SH1000 atl sagA scaH with wildtype PG—red squares SH1000 atl sagA scaH with mutant PG–blue triangles). One mouse in the SH1000 atl sagA scaH and WT PG group reached severity limits at 48 hpi and was culled and has been excluded from analysis. (PDF) [file ppat.1009468.s007.pdf]

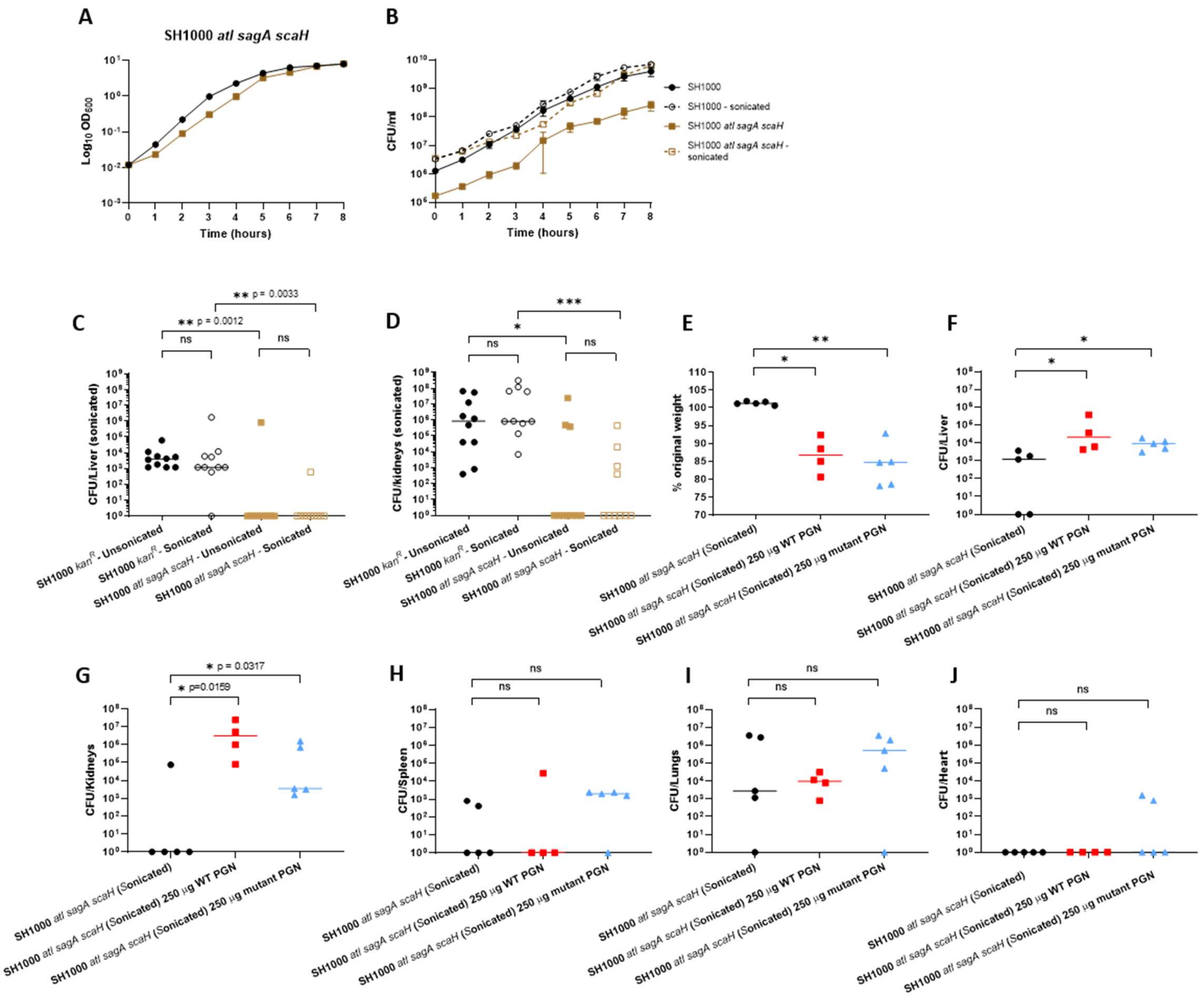

S7 Fig. The role of Atl, SagA and ScaH in *S. aureus* growth and virulence.

(A,B) Growth of parental SH1000 (SJF 682, black circles solid line) or sonicated SH1000 (open black circles and broken line) in TSB compared to SH1000 *atl sagA scaH* (SJF 4611, brown squares) and sonicated SH1000 *atl sagA scaH* (brown open squares, broken lines). Organ homogenates from Fig 7G and 7H were sonicated to get a better representation of bacterial load in the **(C)** livers (p values on graph) and **(D)** kidneys (* p = 0.0364, *** p = 0.0009). Groups were compared using a Kruskal-Wallis test with multiple comparisons (unsonicated SH1000 *kan*^{R} – black circles, sonicated SH1000 *kan*^{R} – open black circles, unsonicated SH1000 *atl sagA scaH* - brown squares and sonicated SH1000 *atl sagA scaH* – open brown squares). Strains and organ homogenates were sonicated for 20 seconds at an amplitude of 5 microns. **(E-J)** Mice (n=5) were injected with approximately 1x10^7 CFU sonicated *S. aureus* SH1000 *atl sagA scaH* (SJF 4611) alone, or with 250 µg SH1000 PG (SJF 682, WT PG) or 250 µg SH1000 *atl sagA scaH* PG (mutant PG). **(E)** Weight loss 72 hpi (** p= 0.0079, * p = 0.0159) and CFUs recovered from **(F)** livers (* p = 0.0159), **(G)** kidneys (p values on graph), **(H)** spleen, **(I)** lungs and **(J)** heart were determined. Groups were compared using a Mann-Whitney U

test. (SH1000 *atl sagA scaH* only – black circles, SH1000 *atl sagA scaH* with wildtype PG - red squares SH1000 *atl sagA scaH* with mutant PG – blue triangles). One mouse in the SH1000 *atl sagA scaH* and WT PG group reached severity limits at 48 hpi and was culled and has been excluded from analysis.
