# Supplementary material for: Staphylococcus aureus cell wall structure and dynamics during host-pathogen interaction
Source: PLoS Pathog. 2021 Mar 31;17(3):e1009468. doi: 10.1371/journal.ppat.1009468 (PMC8041196; doi:10.1371/journal.ppat.1009468)
Supplement: S1 Table — (PDF) [file ppat.1009468.s008.pdf]

Strain	Genotype	Source
SH1000 (SJF 682)	Functional *rsbU*$^+$ derivative of 8325-4	[1]
SH1000 *atl* (SJF 1367)	SH1000 *atl::ery*^{R}	[2]
SH1000 *scaH* (SJF 2109)	SH1000 *scaH::kan*^{R}	[3]
SH1000 *kan*^{R} (SJF 3674)	SH1000 *lysA::kan*^{R} *lysA+*	[4]
SH1000 *sagA* (SJF 4606)	SH1000 *sagA::tet*^{R}	[3]
SH1000 *scaH* (SJF4607)	SH1000 *scaH::tetM*^{R}	[3]
SH1000 *sagB* (SJF 4608)	SH1000 *sagB::kan*^{R}	[3]
SH1000 *atl sagA scaH* (SJF 4611)	SH1000 *atl::spec*^{R} *sagA::tet*^{R} *scaH::tetM*^{R}	[3]
SH1000 *sagA scaH* (SJF 5217)	SH1000 *sagA::tet*^{R} *scaH::kan*^{R}	This study
SH1000 *atl* (SJF 5255)	SH1000 *atl::kan*^{R}	Constructed by Dr B. Salamaga (University of Sheffield)
SH1000 *atl sagA* (SJF 5261)	SH1000 *atl::kan*^{R} *sagA::tet*^{R}	This study
SH1000 *atl scaH* (SJF 5262)	SH1000 *atl::kan*^{R} *scaH::tetM*^{R}	This study
NewHG (SJF 3663)	Newman with *saeS*^{L} allele from strain RN1	[5]
NewHG *kan*^{R} (SJF 3680)	NewHG *lysA::kan*^{R} *lysA+*	[4]
NewHG *tet*^{R} (SJF 3681)	NewHG *lysA::tet*^{R} *lysA+*	[4]
NewHG *sagB* (SJF 4912)	NewHG *sagB::kan*^{R}	This study
NewHG *pbp4* (SJF 5103)	NewHG *pbp4::ery*^{R}	This study
NewHG *tet*^{R} *pbp4* (SJF 5135)	NewHG *lysA::tet*^{R} *lysA+*	This study
NewHG *kan*^{R} *pbp4* (SJF 5136)	NewHG *lysA::kan*^{R} *lysA+ pbp4::ery*^{R}	This study
NewHG *sagB pbp4* (SJF 5147)	NewHG *sagB::kan*^{R} *pbp4::ery*^{R}	This study

S1 Table. *S. aureus* strains used in this study.

References:

1. Horsburgh MJ, Aish JL, White IJ, Shaw L, Lithgow JK, Foster SJ. σB Modulates Virulence Determinant Expression and Stress Resistance: Characterization of a Functional rsbU Strain Derived from Staphylococcus aureus 8325-4. Journal of Bacteriology. 2002;184: 5457–5467. doi:10.1128/JB.184.19.5457-5467.2002

2. Foster SJ. Molecular characterization and functional analysis of the major autolysin of Staphylococcus aureus 8325/4. Journal of Bacteriology. 1995;177: 5723–5725. doi:10.1128/jb.177.19.5723-5725.1995

3. Wheeler R, Turner RD, Bailey RG, Salamaga B, Mesnage S, Mohamad SAS, et al. Bacterial Cell Enlargement Requires Control of Cell Wall Stiffness Mediated by Peptidoglycan Hydrolases. mBio. 2015;6. doi:10.1128/mBio.00660-15

4. McVicker G, Prajsnar TK, Williams A, Wagner NL, Boots M, Renshaw SA, et al. Clonal Expansion during Staphylococcus aureus Infection Dynamics Reveals the Effect of Antibiotic Intervention. PLoS Pathog. 2014;10. doi:10.1371/journal.ppat.1003959

5. Mainiero M, Goerke C, Geiger T, Gonser C, Herbert S, Wolz C. Differential Target Gene Activation by the Staphylococcus aureus Two-Component System saeRS. Journal of Bacteriology. 2010;192: 613–623. doi:10.1128/JB.01242-09
