# Supplementary material for: Staphylococcus aureus cell wall structure and dynamics during host-pathogen interaction
Source: PLoS Pathog. 2021 Mar 31;17(3):e1009468. doi: 10.1371/journal.ppat.1009468 (PMC8041196; doi:10.1371/journal.ppat.1009468)
Supplement: S2 Table — (PDF) [file ppat.1009468.s009.pdf]

Primer	Sequence (5'-3')	Application	Source
Atl_TnINS_F	ACATTTACAGGTGATTTAG CTGTGTTG	Amplifies a region of the *atl* gene. Forward primer	Dr B. Salamaga (University of Sheffield)
Atl_TnINS_R	TCATGATACAGCTAATGAT CGTTCGAC	Amplifies a region of the *atl* gene. Reverse primer	Dr B. Salamaga (University of Sheffield)
psagA_F	TCTCCGATACCAATCCCAG C	Amplifies the *sagA* gene. Forward primer	This study
psagA_R	GCCAATCAAGCGAGTCCAA A	Amplifies the *sagA* gene. Reverse primer	This Study
psagB_F	CCGATCAGATATTTTTCAAA CA	Amplifies the *sagB* gene. Forward primer	[1]
psagB_R	CTTATTCAAATGTTTACTGT CATC	Amplifies the *sagB* gene. Reverse primer	[1]
pscaH_F	AGACGACAGCACTTCAGAC T	Amplifies a region of the *scaH* gene. Forward primer	This study
pscaH_R	AACGGGTGTCTCTACAAGT G	Amplifies a region of the *scaH* gene. Reverse primer	This study
pbp4-1	CTGCAGAAAACTTTATTTTC AAC	Amplifies a region of the *pbp4* gene. Forward primer	Dr K. Wacnik (University of Sheffield)
pbp4-5	TATATAGAACTATCGATAC TAAAC	Amplifies a region of the *pbp4* gene. Reverse primer	Dr K. Wacnik (University of Sheffield)

S2 Table. Oligonucleotides used in this study.

References:

1. Wheeler R, Turner RD, Bailey RG, Salamaga B, Mesnage S, Mohamad SAS, et al. Bacterial Cell Enlargement Requires Control of Cell Wall Stiffness Mediated by Peptidoglycan Hydrolases. mBio. 2015;6. doi:10.1128/mBio.00660-15
