# Supplementary material for: Staphylococcus aureus cell wall structure and dynamics during host-pathogen interaction
Source: PLoS Pathog. 2021 Mar 31;17(3):e1009468. doi: 10.1371/journal.ppat.1009468 (PMC8041196; doi:10.1371/journal.ppat.1009468)
Supplement: S3 Table — Identity of S. aureus muropeptides with numbering system used as a part of this study. The observed retention times, mass and charge states are also noted. (PDF) [file ppat.1009468.s010.pdf]

Muropeptide number	UV retention time (min)	Observed mass (Da)	Charge state (Z)	Calculated neutral mass (Da)	Calculated protonated mass (Da)	Structure
1	24.56	954.37	1	953.40	954.40	GM-Penta(Ala-Gln-Lys-Ala-Gly)
2	29.43-30.59	954.15-954.43	1	953.46	954.46	GM-Tetra(Gln) (G)
3	25.48-31.08	968.07-968.48	1	967.47	968.48	GM-Penta(Gln)
3	26.26	484.57	2	967.47	968.48	GM-Penta(Gln)
4	29.27-32.23	1011.15-1011.47	1	1010.48	1011.48	GM-Tetra(Gln) (GG)
5	30.16-33.77	1025.13-1025.46	1	1024.49	1025.50	GM-Penta(Gln) (G)
5	31.45	513.02-513.06	2	1024.49	1025.50	GM-Penta(Gln) (G)
6	27.65-32.76	1068.10-1068.49	1	1067.50	1068.51	GM-Tetra(Gln) (GGG)
7	31.01	541.59	2	1081.51	1082.52	GM-Penta(Gln) (GG)
7	31.31-32.72	1082.03-1082.40	1	1081.51	1082.52	GM-Penta(Gln) (GG)
8	35.66	1083.12	1	1082.50	1083.51	GM-Penta(Glu) (GG)
9	28.2-34.46	1125.17-1125.5	1	1124.52	1125.53	GM-Tetra(Gln) (GGGG)
9	31.07-32.37	563.05-563.44	2	1124.52	1125.53	GM-Tetra(Gln) (GGGG)
10	32.63-33.46	1139.12-1139.90	1	1138.54	1139.54	GM-Penta(Gln) (GGG)
10	31.6-33.04	570.07-570.80	2	1138.54	1139.54	GM-Penta(Gln) (GGG)
11	30.97-32.83	1182.13-1182.53	1	1181.54	1182.55	GM-Tetra(Gln) (GGGGG)
12	35.05	598.61	2	1195.56	1196.56	GM-Penta(Gln) (GGGG)
12	32.52-34.05	1196.21-1196.48	1	1195.56	1196.56	GM-Penta(Gln) (GGGG)
13	31.94	1239.53	1	1238.54	1239.54	GM-Penta(Ala-Gln-Lys-Ala-Gly) (GGGGG)
14	31.92-34.71	1253.13-1253.61	1	1252.58	1253.59	GM-Penta(Gln) (GGGGG)
14	32.83-34.34	626.99-627.11	2	1252.58	1253.59	GM-Penta(Gln) (GGGGG)
15	34.77	1254.42	1	1253.56	1254.57	GM-Penta(Glu) (GGGGG)
16	43.35-44.5	993.14-993.18	2	1984.94	1985.95	GM-pentapeptide (GGGGG) - M-tetrapeptide(G)
17	40.81-43.7	1066.10-1066.60	2	2131.00	2132.01	GM-pentapeptide (GGGGG) - GM-tetrapeptide
18	41.40-45.03	1094.68-1095.21	2	2188.02	2189.03	GM-pentapeptide (GGGGG) - GM-tetrapeptide(g)
19	42.02	1116.54	2	2231.08	2232.09	GM-pentapeptide(Ala-Gln-Lys-Ala-Gly) (GGGGG) - GM-tetrapeptide(GG)
20	42.53-45.60	1123.21-1123.65	2	2245.04	2246.05	GM-pentapeptide (GGGGG) - GM-tetrapeptide(GG)
21	46.38	1152.14	2	2302.07	2303.07	GM-pentapeptide (GGGGG) - GM-tetrapeptide(GGG)
22	44.18	1173.54	2	2345.09	2346.10	GM-pentapeptide (Ala-Gln-Lys,Ala,Gly) (GGGGG) - GM-tetrapeptide(GGGG)
23	46.38	1180.19	2	2359.09	2360.10	GM-pentapeptide (GGGGG) - GM-tetrapeptide(GGGG)
24	43.03-47.36	1209.06-1209.69	2	2417.49	2418.50	GM-pentapeptide (GGGGG) - GM-tetrapeptide (GGGGG)
24	44.51-45.69	806.75-806.83	3	2417.49	2418.50	GM-pentapeptide (GGGGG) - GM-tetrapeptide (GGGGG)

25	49.05	1118.03	3	3351.36	3352.37	GM-pentapeptide (GGGGG) - GM-tetrapeptide (GGGGG)- GM-tetrapeptide (G)
26	51.88	1733.9	2	3465.53	3466.54	GM-pentapeptide (GGGGG) - GM-tetrapeptide (GGGGG)- GM-tetrapeptide (GGG)
27	56.61	1175.17	3	3522.64	3523.65	GM-pentapeptide (GGGGG) - GM-tetrapeptide (GGGGG)- GM-tetrapeptide (GGGG)
28	52.52-58.60	1193.88-1194.40	3	3579.64	3580.65	GM-pentapeptide (GGGGG) - GM-tetrapeptide (GGGGG)- GM-tetrapeptide (GGGGG)
28	56.16-58.20	895.80-895.85	4	3579.64	3580.65	GM-pentapeptide (GGGGG) - GM-tetrapeptide (GGGGG)- GM-tetrapeptide (GGGGG)
29	67.35-71.38	1582.19-1582.67	3	4744.13	4745.14	GM-tetrapeptide (GGGGG) - GM-tetrapeptide (GGGGG)- GM-tetrapeptide (GGGGG)- GM-tetrapeptide (GGGGG)
29	69.65-73.82	1186.91-1187.26	4	4744.13	4745.14	GM-tetrapeptide (GGGGG) - GM-tetrapeptide (GGGGG)- GM-tetrapeptide (GGGGG)- GM-tetrapeptide (GGGGG)
30	73.04	1186.83	4	4814.21	4815.22	GM-pentapeptide (GGGGG) - GM-tetrapeptide (GGGGG)- GM-tetrapeptide (GGGGG)- GM-tetrapeptide (GGGGG)
31	85.35-90.42	1477.69-1477.83	4	5907.73	5908.74	GM-pentapeptide (GGGGG) - GM-tetrapeptide (GGGGG)- GM-tetrapeptide (GGGGG)- GM-tetrapeptide (GGGGG)- GM-tetrapeptide (GGGGG) (loss of 2 acetyl groups)
31	85.66	1182.63	5	5907.73	5908.74	GM-pentapeptide (GGGGG) - GM-tetrapeptide (GGGGG)- GM-tetrapeptide (GGGGG)- GM-tetrapeptide (GGGGG)- GM-tetrapeptide (GGGGG) (loss of 2 acetyl groups)
32	90.46	1185.14	5	5920.68	5921.69	GM-tetrapeptide (GGGGG) - GM-tetrapeptide (GGGGG)- GM-tetrapeptide (GGGGG)- GM-tetrapeptide (GGGGG)- GM-tetrapeptide (GGGGG)
33	85.02	1484.69-	4	5934.70	5935.71	GM-pentapeptide (GGGGG) - GM-tetrapeptide (GGGGG)- GM-tetrapeptide (GGGGG)- GM-tetrapeptide (GGGGG)- GM-tetrapeptide (GGGG)
34	86.88-88.01	1498.79-1498.89	4	5991.75	5992.76	GM-pentapeptide (GGGGG) - GM-tetrapeptide (GGGGG)- GM-tetrapeptide (GGGGG)- GM-tetrapeptide (GGGGG)- GM-tetrapeptide (GGGGG)
34	89.4	1199.41	5	5991.75	5992.76	GM-pentapeptide (GGGGG) - GM-tetrapeptide (GGGGG)- GM-tetrapeptide (GGGGG)- GM-tetrapeptide (GGGGG)- GM-tetrapeptide (GGGGG)

S3 Table. Muropeptide database.

Identity of *S. aureus* muropeptides with numbering system used as a part of this study. The observed retention times, mass and charge states are also noted.
