# Supplementary material for: Staphylococcus aureus cell wall structure and dynamics during host-pathogen interaction
Source: PLoS Pathog. 2021 Mar 31;17(3):e1009468. doi: 10.1371/journal.ppat.1009468 (PMC8041196; doi:10.1371/journal.ppat.1009468)
Supplement: S4 Table — Identified and measured peaks of (A-C) exponential phase S. aureus, (D-F) stationary phase S. aureus and (G-H) murine infection derived S. aureus. (PDF) [file ppat.1009468.s011.pdf]

A – Exponential phase *S. aureus* muropeptides sample 1							
UV retention time (min)	Observed mass (Da)	Charge state (Z)	Calculated neutral mass (Da)	Calculated protonated mass (Da)	Structure	Area of peak (μAU^2)	% of measured peaks
29.66	1011.43	1	1010.48	1011.48	GM-Tetrapeptide(Gln) (GG)	95354	0.10
30	1068.12	1	1067.50	1068.51	GM-Tetrapeptide(Gln) (GGG)	1755175	1.78
31.69	1025.13	1	1024.50	1025.50	GM-Pentapeptide(Gln) (G)	3597231	3.65
32.57	563.05	2	1124.52	1125.53	GM-Tetrapeptide(Gln) (GGGG)	1141429	1.16
32.89	1082.13	1	1081.51	1082.52	GM-Pentapeptide(Gln) (GG)	155034	0.16
33.25	1139.17	1	1138.54	1139.54	GM-Pentapeptide(Gln) (GGG)	11540220	11.70
42.29	1066.16	2	2131.00	2132.01	GM-pentapeptide (GGGGG) - GM-tetrapeptide	1647840	1.67
44.44	1094.68	2	2188.02	2189.03	GM-pentapeptide (GGGGG) - GM-tetrapeptide(G)	1627802	1.65
45.19	1123.21	2	2245.04	2246.05	GM-pentapeptide (GGGGG) - GM-tetrapeptide(GG)	21840966	22.15
46.38	1180.19	2	2359.09	2360.10	GM-pentapeptide (GGGGG) - GM-tetrapeptide(GGGG)	459587	0.47
47.36	1209.20	2	2417.49	2418.50	GM-pentapeptide (GGGGG) - GM-tetrapeptide (GGGGG)	1293504	1.31
56.45	1193.88	3	3579.64	3580.65	GM-pentapeptide (GGGGG) - GM-tetrapeptide (GGGGG)- GM-tetrapeptide (GGGGG)	19030052	19.30
70.75	1187.01	4	4744.13	4745.14	GM-tetrapeptide (GGGGG) - GM-tetrapeptide (GGGGG)- GM-tetrapeptide (GGGGG)- GM-tetrapeptide (GGGGG)	18510532	18.77
87.19	1498.89	4	5991.75	5992.76	GM-pentapeptide (GGGGG) - GM-tetrapeptide (GGGGG)- GM-tetrapeptide (GGGGG)- GM-tetrapeptide (GGGGG)- GM-tetrapeptide (GGGGG)	15920087	16.14

B – Exponential phase *S. aureus* muropeptides sample 2							
UV retention time (min)	Observed mass (Da)	Charge state (Z)	Calculated neutral mass (Da)	Calculated protonated mass (Da)	Structure	Area of peak (μAU^2)	% of measured peaks
30.54	954.15	1	953.46	954.46	GM-Tetrapeptide(Gln) (G)	2251672	2.41
31.53	1025.43	1	1024.50	1025.50	GM-Pentapeptide(Gln) (G)	4363105	4.66
32.83	1182.47	1	1181.54	1182.55	GM-Tetrapeptide(Gln) (GGGGG)	1789308	1.91
33.71	1253.61	1	1252.58	1253.59	GM-Pentapeptide(Gln) (GGGGG)	14251163	15.24
35.66	1083.12	1	1082.50	1083.51	GM-Pentapeptide(Glu) (GG)	509564	0.54
42.89	1066.17	2	2131.00	2132.01	GM-pentapeptide (GGGGG) - GM-tetrapeptide	1581820	1.69
44.5	993.14	2	1984.94	1985.95	GM-pentapeptide (GGGGG) - M-tetrapeptide(G)	585875	0.63
45.09	1094.68	2	2188.02	2189.03	GM-pentapeptide (GGGGG) - GM-tetrapeptide(G)	1592376	1.70
45.6	1123.21	2	2302.07	2303.07	GM-pentapeptide (GGGGG) - GM-tetrapeptide(GG)	575326	0.62
45.91	1209.28	2	2417.49	2418.50	GM-pentapeptide (GGGGG) - GM-tetrapeptide (GGGGG)	9137041	9.77
57.61	1193.94	3	3579.64	3580.65	GM-pentapeptide (GGGGG) - GM-tetrapeptide (GGGGG)- GM-tetrapeptide (GGGGG)	24094382	25.76
71.38	1582.29	3	4744.13	4745.14	GM-tetrapeptide (GGGGG) - GM-tetrapeptide (GGGGG)- GM-tetrapeptide (GGGGG)- GM-tetrapeptide (GGGGG)	19140300	20.46
86.88	1498.79	4	5991.75	5992.76	GM-pentapeptide (GGGGG) - GM-tetrapeptide (GGGGG)- GM-tetrapeptide (GGGGG)- GM-tetrapeptide (GGGGG)- GM-tetrapeptide (GGGGG)	13665800	14.61

C – Exponential phase *S. aureus* muropeptides sample 3							
UV retention time (min)	Observed mass (Da)	Charge state (Z)	Calculated neutral mass (Da)	Calculated protonated mass (Da)	Structure	Area of peak (μAU^2)	% of measured peaks
28.49	968.10	1	967.47	968.48	GM-Pentapeptide(Gln)	777927	0.83
30.06	1011.47	1	1010.48	1011.48	GM-Tetrapeptide(Gln) (GG)	133649	0.14
30.35	1068.1	1	1067.50	1068.51	GM-Tetrapeptide(Gln) (GGG)	2772561	2.98
31.07	563.06	2	1124.52	1125.53	GM-Tetrapeptide(Gln) (GGGG)	2791406	3.00
31.83	1025.13	1	1024.50	1025.50	GM-Pentapeptide(Gln) (G)	5448645	5.85
32.68	1182.13	1	1181.54	1182.55	GM-Tetrapeptide(Gln) (GGGGG)	1660747	1.78
33	1082.409	1	1081.51	1082.52	GM-Pentapeptide(Gln) (GG)	49127	0.05
33.31	1253.18	1	1252.58	1253.59	GM-Pentapeptide(Gln) (GGGGG)	12676527	13.60
41.69	1066.14	2	2131.00	2132.01	GM-pentapeptide (GGGGG) - GM-tetrapeptide	302122	0.32
43.35	993.18	2	1984.94	1985.95	GM-pentapeptide (GGGGG) - M-tetrapeptide(G)	740070	0.79

44.17	1123.21	2	2302.07	2303.07	GM-pentapeptide (GGGGG) - GM-tetrapeptide(GG)	1168609	1.25
44.47	1209.16	2	2417.49	2418.50	GM-pentapeptide (GGGGG) - GM-tetrapeptide (GGGGG)	7150740	7.67
56.04	1194.23	3	3579.64	3580.65	GM-pentapeptide (GGGGG) - GM-tetrapeptide (GGGGG)- GM-tetrapeptide (GGGGG)	27050239	29.03
69.59	1582.31	3	4744.13	4745.14	GM-tetrapeptide (GGGGG) - GM-tetrapeptide (GGGGG)- GM-tetrapeptide (GGGGG)- GM-tetrapeptide (GGGGG)	17408711	18.68
85.02	1484.69	4	5934.70	5935.70	GM-pentapeptide (GGGGG) - GM-tetrapeptide (GGGGG)- GM-tetrapeptide (GGGGG)- GM-tetrapeptide (GGGGG)- GM-tetrapeptide (GGGG)	13052334	14.01

D – Stationary phase *S. aureus* muropeptides sample 1

UV retention time (min)	Observed mass (Da)	Charge state (Z)	Calculated neutral mass (Da)	Calculated protonated mass (Da)	Structure	Area of peak (μAU^2)	% of measured peaks
31.08	968.16	1	967.47	968.48	GM-Pentapeptide(Gln)	1611130	7.73
33.77	1025.21	1	1024.50	1025.50	GM-Pentapeptide(Gln) (G)	3723043	17.88
34.46	1125.25	1	1124.52	1125.53	GM-Tetrapeptide(Gln) (GGGG)	875593	4.20
35.05	598.61	2	1195.56	1196.56	GM-Pentapeptide(Gln) (GGGG)	4532727	21.77
35.58	1253.25	1	1252.58	1253.59	GM-Pentapeptide(Gln) (GGGGG)	436435	2.10
41.03	1066.19	2	2131.00	2132.01	GM-pentapeptide (GGGGG) - GM-tetrapeptide	718741	3.45
43	1095.20	2	2188.02	2189.03	GM-pentapeptide (GGGGG) - GM-tetrapeptide(G)	338756	1.63
43.29	1209.24	2	2417.49	2418.50	GM-pentapeptide (GGGGG) - GM-tetrapeptide (GGGGG)	1790624	8.60
49.05	1118.03	3	3351.36	3352.37	GM-pentapeptide (GGGGG) - GM-tetrapeptide (GGGGG)- GM-tetrapeptide (G)	4454112	21.39
57.94	1582.26	3	4744.13	4745.14	GM-tetrapeptide (GGGGG) - GM-tetrapeptide (GGGGG)- GM-tetrapeptide (GGGGG)- GM-tetrapeptide (GGGGG)	2341546	11.25

E – Stationary phase *S. aureus* muropeptides sample 2

UV retention time (min)	Observed mass (Da)	Charge state (Z)	Calculated neutral mass (Da)	Calculated protonated mass (Da)	Structure	Area of peak (μAU^2)	% of measured peaks
27.09	968.17	1	967.47	968.48	GM-Pentapeptide(Gln)	1395253	7.80
30.52	954.43	1	953.46	954.46	GM-Tetrapeptide(Gln) (G)	181263	1.01
31.5	1011.15	1	1010.48	1011.48	GM-Tetrapeptide(Gln) (GG)	1586741	8.87
32.8	1025.41	1	1024.50	1025.50	GM-Pentapeptide(Gln) (G)	77104	0.43
33.2	1082.17	1	1081.51	1082.52	GM-Pentapeptide(Gln) (GG)	847040	4.74
33.46	1139.16	1	1138.54	1139.54	GM-Pentapeptide(Gln) (GGG)	201401	1.13
34	1196.24	1	1195.56	1196.56	GM-Pentapeptide(Gln) (GGGG)	215422	1.20
34.34	627.11	2	1252.58	1253.59	GM-Pentapeptide(Gln) (GGGGG)	2060673	11.52
43.7	1066.18	2	2131.00	2132.01	GM-pentapeptide (GGGGG) - GM-tetrapeptide	939232	5.25
45.76	1095.17	2	2188.02	2189.03	GM-pentapeptide (GGGGG) - GM-tetrapeptide(G)	607415	3.40
46.38	1152.14	2	2302.07	2303.07	GM-pentapeptide (GGGGG) - GM-tetrapeptide(GGG)	466105	2.61
46.67	1209.20	2	2417.49	2418.50	GM-pentapeptide (GGGGG) - GM-tetrapeptide (GGGGG)	1401812	7.84
58.8	1194.28	3	3579.64	3580.65	GM-pentapeptide (GGGGG) - GM-tetrapeptide (GGGGG)- GM-tetrapeptide (GGGGG)	3894760	21.78
72.69	1582.25	3	4744.13	4745.14	GM-tetrapeptide (GGGGG) - GM-tetrapeptide (GGGGG)- GM-tetrapeptide (GGGGG)- GM-tetrapeptide (GGGGG)	2845760	15.92
88.01	1498.84	4	5991.75	5992.76	GM-pentapeptide (GGGGG) - GM-tetrapeptide (GGGGG)- GM-tetrapeptide (GGGGG)- GM-tetrapeptide (GGGGG)- GM-tetrapeptide (GGGGG)	1160765	6.49

F – Stationary phase *S. aureus* muropeptides sample 3							
UV retention time (min)	Observed mass (Da)	Charge state (Z)	Calculated neutral mass (Da)	Calculated protonated mass (Da)	Structure	Area of peak (μAU^2)	% of measured peaks
27.9	968.13	1	967.47	968.48	GM-Pentapeptide(Gln)	1066417	7.14
32.23	1011.46	1	1010.48	1011.48	GM-Tetrapeptide(Gln) (GG)	1854402	12.42
32.74	1025.46	1	1024.50	1025.50	GM-Pentapeptide(Gln) (G)	90498	0.61
33.14	1125.17	1	1124.52	1125.53	GM-Tetrapeptide(Gln) (GGGG)	678159	4.54
33.48	1082.49	1	1081.51	1082.52	GM-Pentapeptide(Gln) (GG)	104022	0.70
33.73	1139.17	1	1138.54	1139.54	GM-Pentapeptide(Gln) (GGG)	112005	0.75
34.05	1196.21	1	1195.56	1196.56	GM-Pentapeptide(Gln) (GGGG)	1899417	12.72
34.71	1253.47	1	1252.58	1253.59	GM-Pentapeptide(Gln) (GGGGG)	486675	3.26
42.52	1066.60	2	2131.00	2132.01	GM-pentapeptide (GGGGG) - GM-tetrapeptide	860958	5.77
44.48	1095.13	2	2188.02	2189.03	GM-pentapeptide (GGGGG) - GM-tetrapeptide(G)	561346	3.76
45.32	1209.18	2	2417.49	2418.50	GM-pentapeptide (GGGGG) - GM-tetrapeptide (GGGGG)	1488009	9.97
56.96	1194.18	3	3579.64	3580.65	GM-pentapeptide (GGGGG) - GM-tetrapeptide (GGGGG)- GM-tetrapeptide (GGGGG)	4069909	27.26
71.27	1582.19	3	4744.13	4745.14	GM-tetrapeptide (GGGGG) - GM-tetrapeptide (GGGGG)- GM-tetrapeptide (GGGGG)- GM-tetrapeptide (GGGGG)	1658858	11.11

G – Murine kidney derived *S. aureus* muropeptides sample 1							
UV retention time (min)	Observed mass (Da)	Charge state (Z)	Calculated neutral mass (Da)	Calculated protonated mass (Da)	Structure	Area of peak (μAU^2)	% of measured peaks
30.59	954.15	1	953.46	954.46	GM-Tetrapeptide(Gln) (G)	127005	2.68
31.45	513.02	1	1024.50	1025.50	GM-Pentapeptide(Gln) (G)	421883	8.90
32.37	563.44	2	1124.52	1125.53	GM-Tetrapeptide(Gln) (GGGG)	155394	3.28
32.69	1082.03	1	1081.51	1082.52	GM-Pentapeptide(Gln) (GG)	88455	1.87
33.04	570.07	1	1138.54	1139.54	GM-Pentapeptide(Gln) (GGG)	67071	1.42
33.34	627.08	2	1252.58	1253.59	GM-Pentapeptide(Gln) (GGGGG)	896838	18.93
42.63	1066.30	2	2131.00	2132.01	GM-pentapeptide (GGGGG) - GM-tetrapeptide	151557	3.20
44.78	1094.98	2	2188.02	2189.03	GM-pentapeptide (GGGGG) - GM-tetrapeptide(G)	200563	4.23
45.4	1123.45	2	2245.04	2246.05	GM-pentapeptide (GGGGG) - GM-tetrapeptide(GG)	321133	6.78
45.75	1209.48	2	2417.49	2418.50	GM-pentapeptide (GGGGG) - GM-tetrapeptide (GGGGG)	1066850	22.51
58.6	1194.30	3	3579.64	3580.65	GM-pentapeptide (GGGGG) - GM-tetrapeptide (GGGGG)- GM-tetrapeptide (GGGGG)	780868	16.48
73.04	1186.83	4	4814.21	4815.22	GM-tetrapeptide (GGGGG) - GM-tetrapeptide (GGGGG)- GM-tetrapeptide (GGGGG)- GM-tetrapeptide (GGGGG)	460963	9.73

H – Murine kidney derived *S. aureus* muropeptides sample 2							
**UV retention time (min)**	**Observed mass (Da)**	**Charge state (Z)**	**Calculated neutral mass (Da)**	**Calculated protonated mass (Da)**	**Structure**	**Area of peak (μAU^2)**	**% of measured peaks**
29.43	954.32	1	953.46	954.46	GM-Tetrapeptide(Gln) (G)	350575	6.40
30.84	1025.35	1	1024.50	1025.50	GM-Pentapeptide(Gln) (G)	557820	10.19
31.76	1182.43	1	1181.54	1182.55	GM-Tetrapeptide(Gln) (GGGGG)	905240	16.53
32.52	1196.48	1	1195.56	1196.56	GM-Pentapeptide(Gln) (GGGG)	390446	7.13
33.15	1253.39	1	1252.58	1253.59	GM-Pentapeptide(Gln) (GGGGG)	970851	17.73
45.14	1209.53	2	2417.49	2418.50	GM-pentapeptide (GGGGG) - GM-tetrapeptide (GGGGG)	1314932	24.01
56.36	1194.4	3	3579.64	3580.65	GM-pentapeptide (GGGGG) - GM-tetrapeptide (GGGGG)- GM-tetrapeptide (GGGGG)	985898	18.00

S4 Table. Raw integration results.

Identified and measured peaks of **(A-C)** exponential phase *S. aureus*, **(D-F)** stationary phase *S. aureus* and **(G-H)** murine infection derived *S. aureus.*
